# Analysis of the RelA:CBP/p300 Interaction Reveals Its Involvement in NF-κB-Driven Transcription

**DOI:** 10.1371/journal.pbio.1001647

**Published:** 2013-09-03

**Authors:** Sulakshana P. Mukherjee, Marcelo Behar, Harry A. Birnbaum, Alexander Hoffmann, Peter E. Wright, Gourisankar Ghosh

**Affiliations:** 1 Department of Chemistry and Biochemistry, University of California, San Diego, La Jolla, California, United States of America; 2 Department of Molecular Biology and The Skaggs Institute for Chemical Biology, The Scripps Research Institute, La Jolla, California, United States of America; 3 Signaling Systems Laboratory, University of California, San Diego, La Jolla, California, United States of America; Max Planck Institute for Immunobiology, Germany

## Abstract

NF-κB plays a vital role in cellular immune and inflammatory response, survival, and proliferation by regulating the transcription of various genes involved in these processes. To activate transcription, RelA (a prominent NF-κB family member) interacts with transcriptional co-activators like CREB-binding protein (CBP) and its paralog p300 in addition to its cognate κB sites on the promoter/enhancer regions of DNA. The RelA:CBP/p300 complex is comprised of two components—first, DNA binding domain of RelA interacts with the KIX domain of CBP/p300, and second, the transcriptional activation domain (TAD) of RelA binds to the TAZ1 domain of CBP/p300. A phosphorylation event of a well-conserved RelA(Ser276) is prerequisite for the former interaction to occur and is considered a decisive factor for the overall RelA:CBP/p300 interaction. The role of the latter interaction in the transcription of RelA-activated genes remains unclear. Here we provide the solution structure of the latter component of the RelA:CBP complex by NMR spectroscopy. The structure reveals the folding of RelA–TA2 (a section of TAD) upon binding to TAZ1 through its well-conserved hydrophobic sites in a series of grooves on the TAZ1 surface. The structural analysis coupled with the mechanistic studies by mutational and isothermal calorimetric analyses allowed the design of RelA-mutants that selectively abrogated the two distinct components of the RelA:CBP/p300 interaction. Detailed studies of these RelA mutants using cell-based techniques, mathematical modeling, and genome-wide gene expression analysis showed that a major set of the RelA-activated genes, larger than previously believed, is affected by this interaction. We further show how the RelA:CBP/p300 interaction controls the nuclear response of NF-κB through the negative feedback loop of NF-κB pathway. Additionally, chromatin analyses of RelA target gene promoters showed constitutive recruitment of CBP/p300, thus indicating a possible role of CBP/p300 in recruitment of RelA to its target promoter sites.

## Abstract 2

A structural and functional study delineates how the interaction between NF-κB subunit RelA and co-activator CBP/p300 helps drive transcription of NF-κB target genes.

## Author Summary

The NF-κB family of transcription factors regulate the expression of numerous genes involved in the immune response, cell survival, differentiation, and proliferation. The interaction of the RelA subunit of NF-κB with the general co-activator protein CBP/p300 is vital for RelA-dependent gene transcription. Although the recruitment of RelA to its cognate genomic κB sites for target gene activation is well-established, the involvement of CBP/p300 in this process remains unclear. Through our structure/function-based approach we provide the molecular and functional details of the RelA:CBP/p300 interaction and its contribution to the regulation of distinct subsets of target genes. We also show that disruption of this interaction deregulates the NF-κB pathway by interfering with its negative feedback loop. Furthermore, our study indicates a possible reciprocal role for CBP/p300 in the recruitment of RelA to its target gene promoters.

## Introduction

The NF-κB family of inducible transcription factors has emerged as a major player in immune response [1], activating a plethora of immunoregulatory genes upon stimulation. In vertebrates, the family is comprised of five members, namely, RelA (also known as p65), RelB, c-Rel, p50, and p52. They exist in various combinations of homo- and hetero-dimers, with RelA:p50 being the most abundant NF-κB dimer present in the cell. All of the family members share a structurally conserved N-terminal rel homology region (RHR), which is responsible for DNA binding and also contains the dimerization domain (DD). RelA, RelB, and c-Rel distinguish themselves from p50 and p52 by possessing the transcriptional activation domain (TAD), which is vital for the transcriptional regulation of the NF-κB-regulated genes. In unstimulated cells, NF-κB is maintained in a latent state in the cytoplasm by the family of Inhibitor-κB (IκB) proteins, which strongly bind to the RHR domain of NF-κB dimers, thereby masking its nuclear localization signal. Upon stimulation, a cascade of signaling events led to the degradation of the IκB proteins, thus releasing the NF-κB dimers, which then enter the nucleus to regulate the transcription of NF-κB-dependent genes. In the nucleus, NF-κB activates transcription by binding to its cognate κB sites in the promoters/enhancers of its target genes [2],[3]. However, mere binding of the NF-κB to its cognate κB sites does not ensure transcriptional initiation, and further requires the assembly of the “basal transcription machinery” on the transcription start site. Assembly of the transcription initiation complex requires NF-κB to interact with the mediator complex, various transcriptional adaptors, and co-activator proteins like CREB-binding protein (CBP) and its paralog p300 [4]–[7]. CBP/p300 are general transcriptional co-activators that help NF-κB bridge with the basal transcription machinery [8]. CBP/p300 interact with a large array of transcription factors to integrate multiple cellular signaling pathways [9],[10] and also possess chromatin-remodeling capabilities owing to their histone- and transcription factor–acetylating properties.

To date, it is widely believed that in the nucleus NF-κB recruits CBP/p300 to its target promoter sites, and in this process it must compete with various other cellular transcription factors for the limiting amounts of CBP/p300 [11]. However, in recent genome-wide analyses of gene promoters/enhancers using ChIP-chip and ChIP-seq technology, p300 has emerged as a prominent marker for enhancers [12]. p300 is also pre-loaded in most of the promoters and enhancers of NF-κB-regulated genes marked with histone-H3 lysine 4 trimethyl (H3K4me3) and histone H3 lysine 4 monomethyl (H3K4me1), respectively, in unstimulated THP-1 and HeLa cells [13]. In light of these new findings, the question arises that if p300 is already preloaded on the promoter/enhancer regions of the NF-κB target genes, then what is the role of NF-κB:CBP/p300 binding in the NF-κB activated transcription.

**Figure 1:**
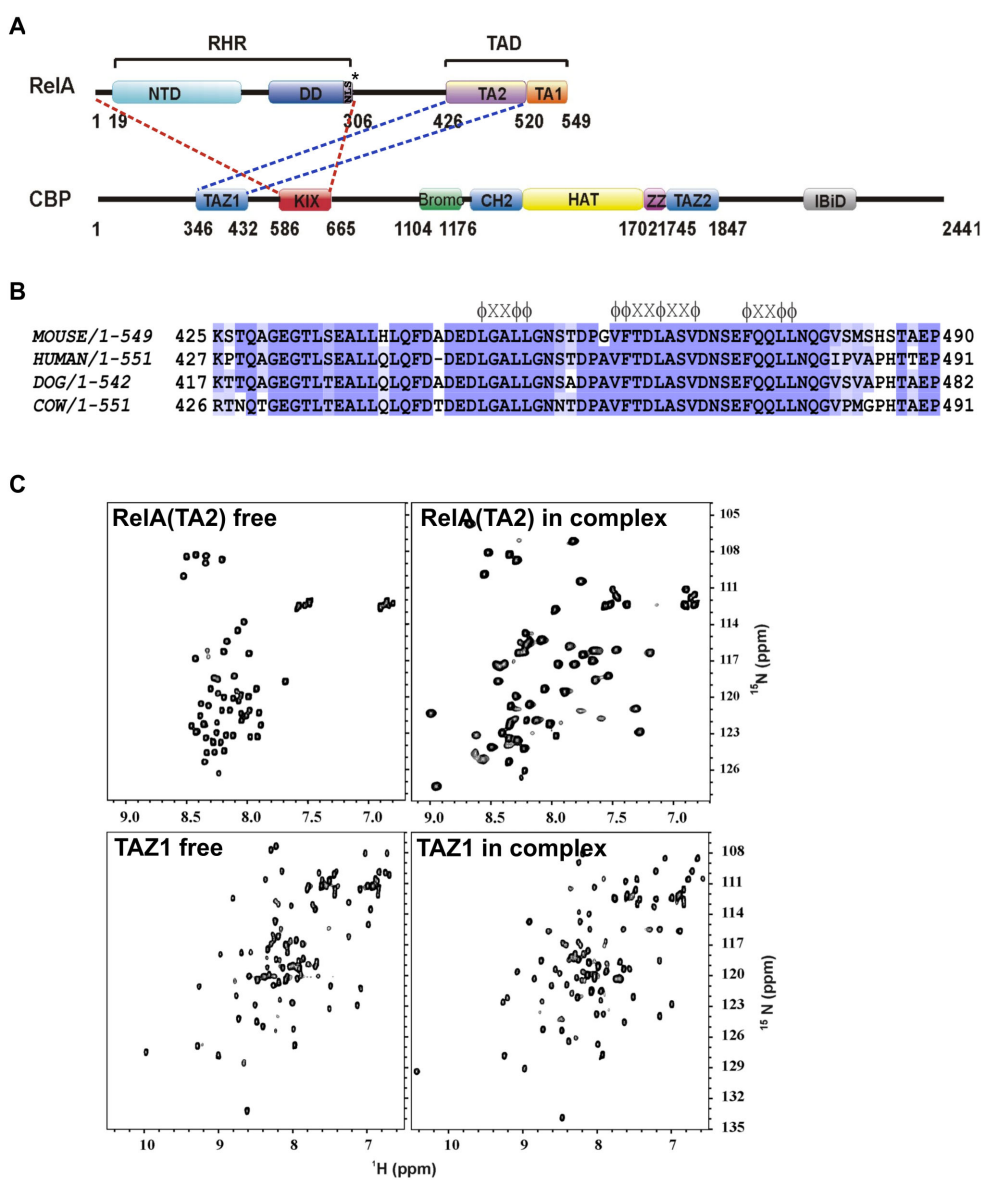
The intrinsically disordered RelA transactivation domain folds on the TAZ1 domain of CBP. (A) Schematic of domain organization of RelA and CBP. The approximate boundaries for CBP were taken from the uniprotkB database (primary accession number P45481). * denotes the nuclear localization signal (NLS). (B) Sequence alignment of mammalian RelA–TA2 region. The ψXXψψ motifs and the ψψXXψXXψ sequence are marked over the sequence, where ψ is a hydrophobic residue and X can be any residue. The sequence alignment was done using Jalview Version 2 software [77]. (C) [^15^N-^1^H]-HSQC spectra of ^15^N labeled free RelA–TA2 (425–490) (upper left panel), ^15^N labeled RelA–TA2 in complex with unlabeled TAZ1 (340–349) (upper right panel), ^15^N labeled free TAZ1 (lower left panel), and ^15^N labeled TAZ1 in complex with unlabeled RelA–TA2 (lower right panel). [^15^N-^1^H]-HSQC spectra with narrow dispersion in the ^1^H dimension as observed for the free RelA–TA2 fragment indicate unstructured protein.

Among the NF-κB proteins, RelA is the most potent, ubiquitously expressed and well-studied family member. It contains a TAD, which can be further divided into two regions, TA1 and TA2, as depicted in Figure 1A [14]. Although the activation of the NF-κB pathway is extensively studied [15], mechanistic details of the transcription initiation process by NF-κB at the promoter site are limited [16]. This can partly be attributed to the lack of any structural information and limited overall knowledge of RelA–TAD [17],[18]. The interaction of RelA with CBP/p300 represents a key step in the initiation of transcription of a subset of RelA-activated genes [19]. RelA interacts with CBP/p300 in a bipartite manner—the RHR domain of RelA contacts the KIX domain of CBP/p300 and the TAD of RelA contacts the TAZ1 (also known as the CH1) domain of CBP/p300 [20]. The former interaction requires RelA-phosphorylation at a well-conserved Ser276 residue. Although both the RHR:KIX and TAD:TAZ1 interactions contribute to transcriptional activation of RelA-dependent genes, phosphorylation of Ser276 is considered critical for the RelA:CBP/p300 interaction [20]. It remains unclear whether the interaction between the RelA–TAD and the TAZ1 domain of CBP/p300 can activate transcription of RelA-target genes independent of RelA(Ser276) phosphorylation or vice versa. In addition to CBP/p300, RelA also interacts with a number of other proteins for the transcriptional activation of its target genes through its TAD, and hence a detailed knowledge of the TAD:TAZ1 binding is a necessary prelude to any mutations in the RelA–TAD, which can selectively abrogate RelA:CBP/p300 interaction [16].

As an initial step towards elucidating the role of NF-κB:CBP/p300 interaction in transcription of NF-κB-regulated genes, we determined the solution structure of the mouse RelA–TA2 (a subdomain of TAD) in complex with the TAZ1 domain of mouse CBP. The structure reveals the high affinity binding of RelA–TA2 through its well-conserved hydrophobic sites (Figure 1B) in a series of grooves on the TAZ1 surface. The structure enabled us to design point mutants of RelA, which selectively abrogated the RelA–TAD:CBP–TAZ1 binding, thereby allowing us to gain detailed insight into the molecular determinants of the RelA:CBP/p300 interaction. Using these RelA mutants defective in CBP/p300 binding, we performed a genome-wide analysis of the genes influenced by the RelA:CBP/p300 interaction to confirm the differential role of the two interaction sites on the RelA regulated transcriptome. We found genes like *nfkbia* and *ptgs2*, which were previously considered independent of RelA:CBP/p300 interaction [19], were actually dependent on it. The dependence of *nfkbia* expression on RelA:CBP/p300 interaction explains how this interaction regulates the temporal profile of nuclear NF-κB (nNF-κB) following TNFα stimulation. Furthermore, we revisited the model of CBP/p300 recruitment by RelA using Chromatin Immunoprecipitation (ChIP) assay. Our study confirmed that CBP/p300 is preloaded on the promoter regions of our set of RelA target genes, thus indicating that RelA:CBP/p300 interaction might be vital for recruitment of RelA to its target promoter sites.

## Results

### Mapping of the CBP–TAZ1 Binding Site on RelA–TAD

The RelA–TAD:CBP–TAZ1 interaction was previously mapped to RelA(477–504) region [20] (Figure 1A). We found only a weak interaction between this RelA fragment and TAZ1 as compared to the whole RelA–TAD [RelA(425–549)] and therefore scanned the entire TAD for TAZ1 binding using GST pull-down assays (Figure S1). A RelA construct spanning residues Lys425–Pro490 (in the TA2 region) emerged as the minimal fragment with maximal affinity to TAZ1. This fragment (henceforth referred to as RelA–TA2) was also optimal for structural analysis by NMR spectroscopy (Figure S1B,C). This highly acidic RelA–TA2 fragment is unstructured in its free state, as observed from the narrow dispersion of peaks in the [^15^N-^1^H]–HSQC spectrum, but exhibits a well-dispersed spectrum indicative of a folded structure when in complex with TAZ1 (Figure 1C).

### Structure of CBP–TAZ1 in the RelA–TA2:CBP–TAZ1 Complex

**Figure 2:**
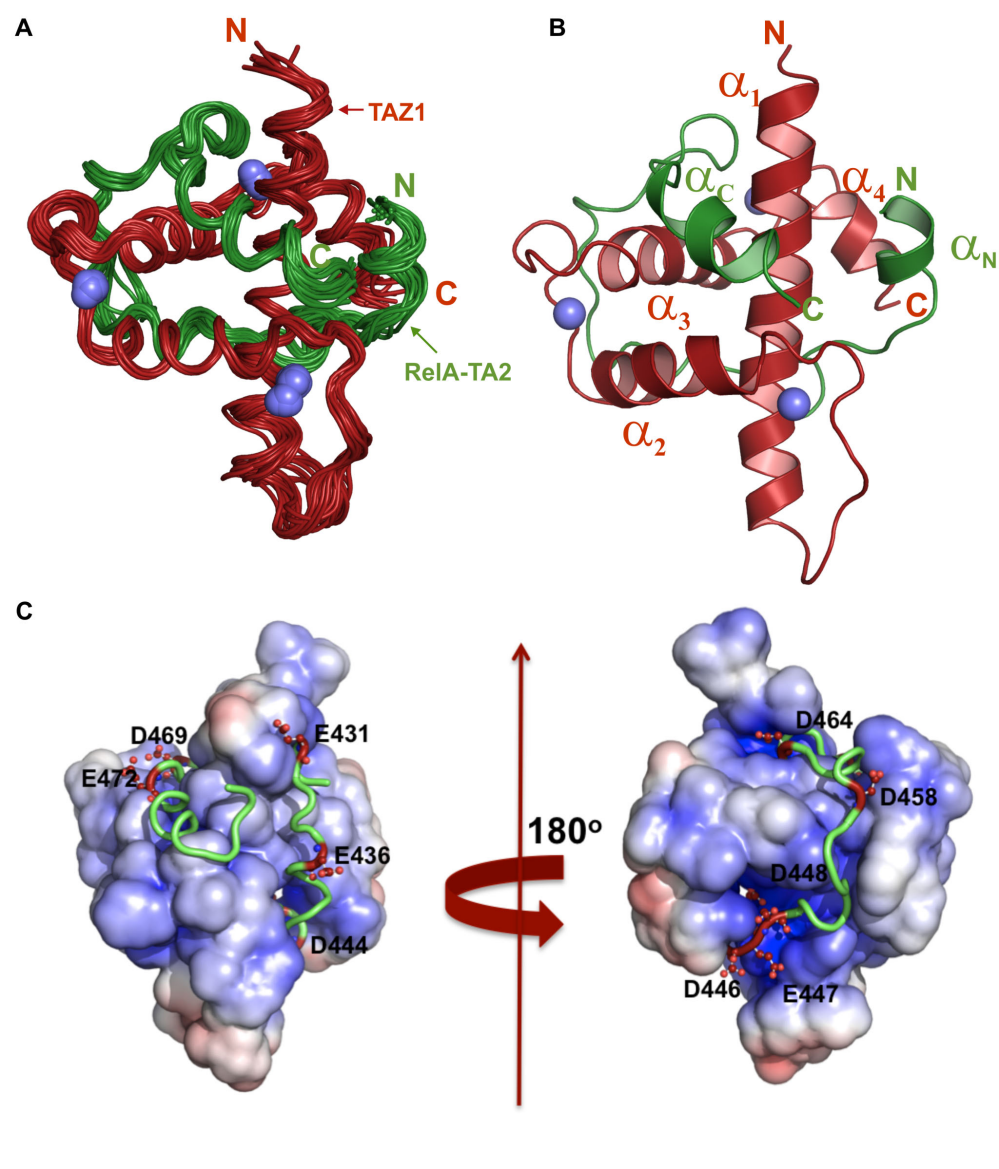
NMR structure of the RelA–TA2:CBP–TAZ1 complex. RelA–TA2 is shown in green and TAZ1 in red. The three Zn^2+^ ligands are depicted in blue. The ordered region of the RelA fragment (Leu434–Val481) and TAZ1 (Ala345–Asp437) is depicted unless otherwise mentioned. (A) Twenty superimposed lowest energy NMR structures of RelA–TA2:CBP–TAZ1 complex. (B) Cartoon depiction of the lowest energy model of RelA–TA2:CBP–TAZ1 complex. (C) Electrostatic potential of solvent accessible surface of TAZ1 in complex with RelA–TA2. The positive potential is shown in blue and the negative in red. RelA–TA2 is shown as green ribbon with its acidic residues shown as red ball-and-sticks. RelA fragment (Gly430–Met483) is depicted in this figure.

The structure of the complex between RelA–TA2 (residues 425–490) and CBP–TAZ1 (residues 340–439) was determined using distance and dihedral angle restraints derived from heteronuclear NMR experiments (see Table 1 for NMR statistics). TAZ1 is a scaffolding domain that interacts with intrinsically disordered TADs of various transcription factors, which fold upon complex formation [21]–[23]. The overall structure of TAZ1 in the complex (Figure 2) resembles that of TAZ1 in its free state (RMSD 1.6 Å) and in complex with other protein targets like HIF-1α (RMSD 1.8 Å), CITED2 (1.4 Å), and STAT2 (RMSD 1.3 Å) [21],[23]–[25]. As observed in our structure, the TAZ1 fold comprises four α-helices (α_1_, α_2_, α_3_, and α_4_) arranged in a roughly tetrahedral shape and is stabilized by three zinc atoms, each of which is bound to three Cys residues and one His residue. A prominent surface feature of the TAZ1 domain is a series of interlinked hydrophobic grooves that bind intrinsically disordered TADs (Figure S2).

**Table 1:** NMR and refinement statistics for protein structures.

Statistics	RelA(TA2):TAZ1
NMR distance and dihedral constraints	
Distance constraints	
Total NOE	2,012
Intra-residue	684
Inter-residue	
Sequential (|i – j| = 1)	448
Medium-range (2< = |i – j|< = 4)	441
Long-range (|i – j|> = 5)	186
Intermolecular	253
Hydrogen bonds	0
Total dihedral angle restraints	
φ	110
ψ	109
Structure statistics	
Violations (mean and s.d.)	
Distance constraints (Å)	0.08±0.02
Dihedral angle constraints (°)	0.62±0.03
Max. dihedral angle violation (°)	0.64
Max. distance constraint violation (Å)	0.21
Deviations from idealized geometry	
Bond lengths (Å)	0.0103±0.0007
Bond angles (°)	2.657±0.052
Average pairwise r.m.s. deviation** (Å)	
Heavy	1.208
Backbone	0.722

** Pairwise r.m.s. deviation was calculated among 20 refined structures for the regions (TAZ1): K349–A372, T386–N434; (RelA(TA2): L434–V481.

The Ramachandran plot of the RelA(TA2):TAZ1 structure, 92.0% of the backbone dihedral angles were in the most favorable region, 7.9% in the additionally allowed region, 0% in the generously allowed region, and 0% in the disallowed region.

### Structure of RelA–TA2 in the RelA–TA2:CBP–TAZ1 Complex

In the RelA–TA2:TAZ1 structure, the N- and C-terminal regions of the RelA–TA2 fragment used for structural analysis (Lys425–Thr433 and Ser482–Pro490, respectively) are dynamically disordered, with zero or negative [^1^H]-^15^N heteronuclear NOEs (Figure S3A), and hence are omitted from the structures shown in Figure 2A,B. Our structure shows that RelA–TA2, essentially the Leu434–Val481 region, entirely wraps around TAZ1 in a predominantly extended conformation by docking itself through its well-conserved bulky hydrophobic residues into the interlinked hydrophobic grooves of TAZ1 (Figure 2 and Figure S4A). The C-terminal region (Glu471–Asn477) of RelA–TA2 folds into one short helix α_C_ that is anchored to the hydrophobic pocket formed by packing of α_1_, α_2_, and α_3_ helices of TAZ1 (Figure 2B). Likewise, the N-terminal region (Leu434–Leu439) of RelA–TA2 also forms a short helix α_N_, which is docked into the shallow hydrophobic groove formed at the junction of α_1_ and α_4_ of TAZ1. The α_N_ helix is dynamically disordered with only about 30% of the helical population, as calculated from the magnitude of the C-alpha and carbonyl secondary chemical shifts [26] (Figure S3B), and the small value of the [^1^H]-^15^N heteronuclear NOE (∼0.3–0.5 for the α_N_ residues) confirms that this region is highly flexible on the nanosecond time scale (Figure S3A). RelA–TA2 also forms two additional helical turns, Leu449–Leu452 and Leu465–Val468, which make hydrophobic contacts in the α_1_–α_2_–α_3_ and α_1_–α_3_ interfaces of TAZ1, respectively. The hydrophobic interactions are complemented by electrostatic interactions between the highly acidic RelA–TA2 region and the strongly electropositive surface of TAZ1. The region from Asp444–Asp448 in RelA–TA2 is particularly acidic, with four out of five residues being negatively charged, and passes through a deep cleft in the TAZ1 surface that is lined with basic residues (Lys366, Arg368, Arg369, Lys419, Lys438, Arg439) (Figure 2C). Our structural analysis was further corroborated by ITC experiments carried out at two different salt concentrations to study the electrostatic contribution of the binding event. Increasing the NaCl concentration from 50 to 150 mM weakened RelA–TA2:TAZ1 binding by 4-fold, consistent with a significant electrostatic contribution to binding from the complementary charges on TAZ1 and the RelA–TA2 region (Table 2).

**Table 2:** Summary of dissociation constants and thermodynamic parameters obtained from ITC experiments.

RelA Peptides	Conc. NaCl (mM)	Dissociation Constants (nM)	ΔH (kcal/mol)	TΔS (kcal/mol)
425–508 wt	50	57.0±3.2	−5.9±0.1	3.9±0.1
425–508 (S467D)	50	41.2±0.3	−6.5±0.1	3.5±0.1
425–508 wt	150	244.4±11.3	−7.2±0.1	1.8±0.1
441–508 wt	150	386.8±33.4	−8.3±0.4	0.5±0.5
425–508 (L449A)	150	1,604.5±194.1	−5.4±0.3	2.5±0.3
425–508 (L465A)	150	1,706.8±434.5	−2.4±0.3	5.5±0.5
425–508 (F473A)	150	NB*	NB*	NB*
425–508 (S467D)	150	173.9±7.2	−7.8±0.1	1.4±0.1

* NB = No Binding under experimental conditions.

Thermodynamic parameters of binding are mentioned along with standard deviation (s.d.).

### RelA–TA2 Anchors on CBP–TAZ1 Through Multiple Hydrophobic Residues

**Figure 3:**
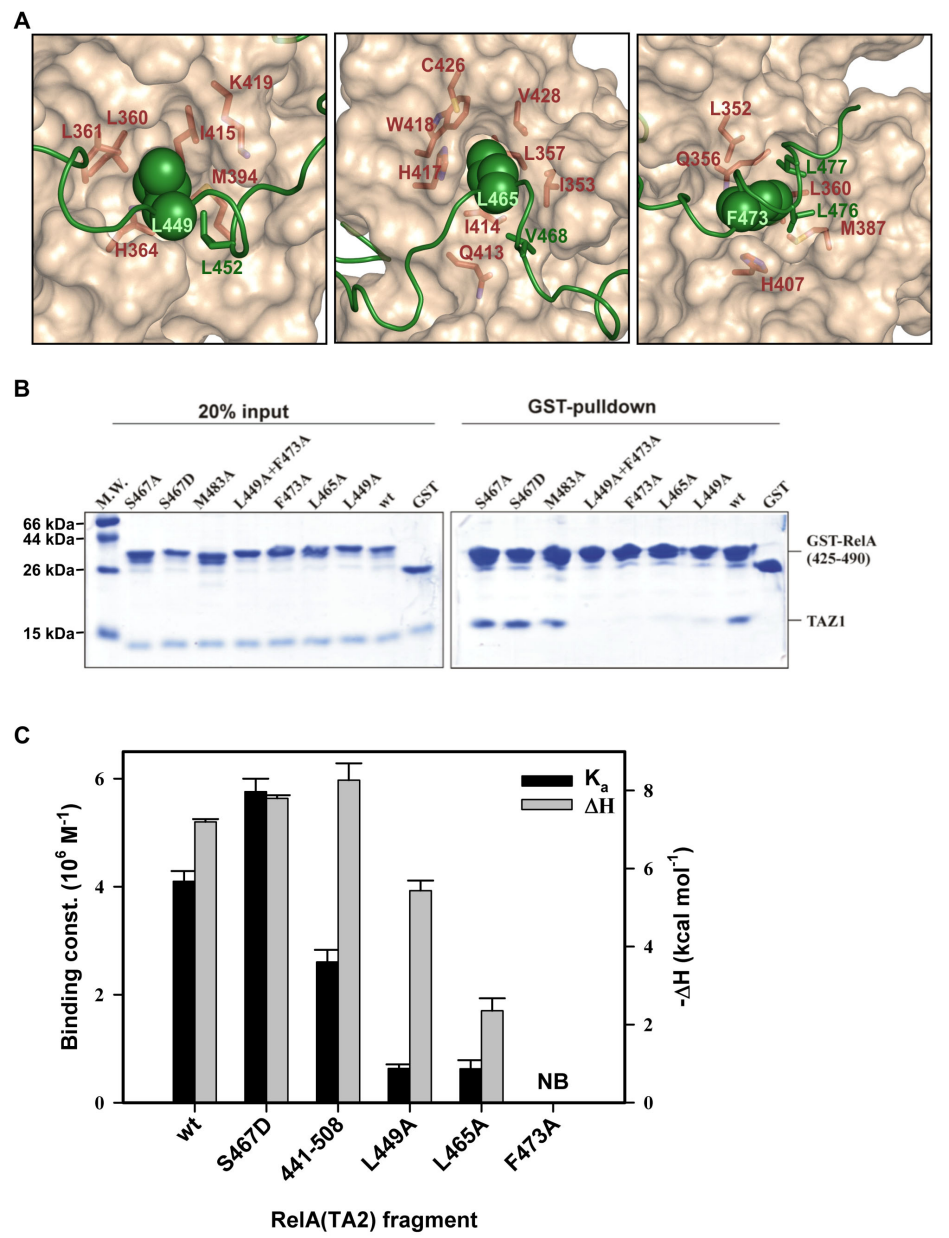
RelA–TA2 docks itself onto a series of interlinked hydrophobic grooves of TAZ1 through a number of hydrophobic residues, which serve as anchor points. Disruption of any of the anchor points leads to the destabilization of the whole binding architecture of the complex. ∼2,800 Å^2^ of RelA–TA2 surface area are buried upon formation of RelA–TA2:TAZ1 complex. (A) Leu449 (left panel), Leu465 (middle panel), and Phe473 (right panel) residues of RelA–TA2 (shown as green spheres) buried into each face of the interlinked hydrophobic grooves of TAZ1. Residues of TAZ1 making contacts with the above mentioned hydrophobic residues of RelA are shown as red sticks. The other hydrophobic residues of RelA–TA2—namely, Leu452, Val468, Leu476, and Leu477—making vital contacts with TAZ1 are depicted as green sticks. (B) GST-pulldown experiments performed on the RelA–TA2 mutants binding to TAZ1. RelA fragment (Lys425–Pro490) with N-terminal GST-tag was used as the wild-type protein. All the mutations were made using this construct as a template. (C) Vertical bar chart of the association constants (left vertical axis, black bars) and enthalpy change (right vertical axis, grey bars) obtained from the ITC binding isotherms of the RelA–TA2:TAZ1 interaction corresponding to the respective RelA–TA2 wt and mutants (see Figure S5). The Leu449Ala and Leu465Ala mutants in RelA–TA2 lead to diminished association constants accompanied with decreased negative enthalpy change. The Phe473Ala mutant showed no binding (NB).

The structure of the RelA–TA2:TAZ1 complex revealed that several hydrophobic residues in RelA–TA2 mediate binding within the exposed hydrophobic grooves of TAZ1. The RelA–TA2 amino acid residues, which extensively interact with the hydrophobic pockets of TAZ1, belong to two ψXXψψ motifs and a ψψXXψXXψ sequence (Figure 3A), where ψ is a bulky hydrophobic residue (Leu, Val or Phe in RelA–TA2) and X can be any residue (Figure 1B). The ψXXψψ motif is a generalization of the LXXLL motifs that are known to mediate protein–protein interactions [27]. Furthermore, Val481, Phe443, and the Leu residues belonging to the α_N_ helix of RelA–TA2 also contribute to complex formation as observed from the intermolecular NOEs (Figure S4B).

To investigate the role of the ψXXψψ motifs, we substituted alanine for Leu449 and Phe473 of RelA, each of which represents the first ψ residue of the motif. Both Leu449 and Phe473 are deeply buried in the molecular interface and participate in extensive hydrophobic interactions (Figures 1B and 3A). We also replaced the third ψ residue of ψψXXψXXψ sequence with Ala–Leu465Ala (Figure 3A, middle panel and Figure S4A). All three RelA mutants showed diminished to completely abrogated TAZ1 binding in the GST-pulldown assays (Figure 3B) and isothermal calorimetric (ITC) experiments (Figure S5, Table 2, Figure 3C), confirming the critical role of hydrophobic interactions in stabilizing the complex. The Leu449Ala and Leu465Ala substitutions each led to a 7-fold decrease in the TAZ1 binding affinity (Figure 3C and Table 2). As seen in the structure, the side chain of Leu465 is fully buried into a deep narrow hydrophobic pocket formed by Ile353, Leu357, Gln413, Ile414, His417, Trp418, Cys426, and Val428 of TAZ1 and Val468 of RelA (Figure 3A, middle panel and Figure S4A). This interaction contributes significantly to enthalpic stabilization of the complex, since the Leu465Ala substitution greatly decreases the enthalpic contribution to TAZ1 binding (Figure 3C). Leu449 binds in a shallower hydrophobic pocket, and accordingly substitution by alanine causes far less enthalpic destabilization (Figure 3A, left panel). The Phe473Ala mutation led to a complete loss of TAZ1 binding. In the structure, the side chains of Phe473, Leu476, and Leu477 (all the ψ residues of this ψXXψψ motif) are accommodated in the hydrophobic groove of TAZ1 (Figure 3A, right panel and Figure S4A). This arrangement is destabilized in the Phe473Ala mutant, leading to complete loss of TAZ1 binding. We speculate that a similar effect would be observed for Leu476Ala and Leu477Ala mutants.

Apart from the above-mentioned residues of RelA–TA2, the contacts between TAZ1 and the disordered α_N_ helix of RelA–TA2 contribute slightly to formation of the RelA–TA2:TAZ1 complex (Figure S4B). N-terminal truncation to remove residues Lys425–His440, thereby eliminating the entire α_N_ helix, decreases the affinity by only 1.6-fold (Figure 3C and Table 2). Likewise, deletion of residues Ser486–Pro490 from the disordered C-terminal end of RelA had no effect on TAZ1 binding (Figure S1D). However, further truncation to remove Val481–His485 greatly impaired binding by eliminating hydrophobic contacts between Val481 of RelA(TA2) and the side chains of Leu359, Leu381, and Pro382 in TAZ1. Replacement of Met483 by alanine had no effect on binding (Figure 3B), confirming that RelA residues beyond Val481 do not contribute to the interaction.

In summary, our structure shows that RelA–TA2 spirals through the exposed hydrophobic pockets of TAZ1 and anchors itself on TAZ1 at a number of points. As observed from the mutation studies, disruption of any of these anchoring residues of RelA–TA2 leads to the destabilization of the entire binding architecture of the entire complex.

### Possible Role of Phosphorylation in RelA–TA2:CBP–TAZ1 Binding

The RelA–TA2 region also contains a well-conserved Ser467 (Ser468 in human RelA), which is a known phosphorylation site. Depending upon the stimulus, this site can be phosphorylated by GSK3β, IKKε, or IKKβ and plays a critical role in transcriptional regulation of NF-κB dependent genes [28]–[31]. Ser467 resides in the ψψXXψXXψ sequence of RelA–TA2 (Figure 1B), and in the complex it is close to the cluster of basic residues at the N-terminus of helix α_1_ of TAZ1. Our ITC experiments show that a phosphomimetic mutant, RelA(Ser467Asp), binds TAZ1 with about 1.4-fold higher affinity than wild type RelA–TA2 and exhibits the same dependence on ionic strength, suggesting an enhanced but still nonspecific electrostatic interaction (Table 2 and Figure 3C). Thus, the increased negative charge of phosphoserine would likely enhance TAZ1 binding even further over nonphosphorylated RelA.

### Comparative Binding Analysis of RelA–RHR:CBP–KIX and RelA–TA2:CBP–TAZ1 Interactions

**Figure 4:**
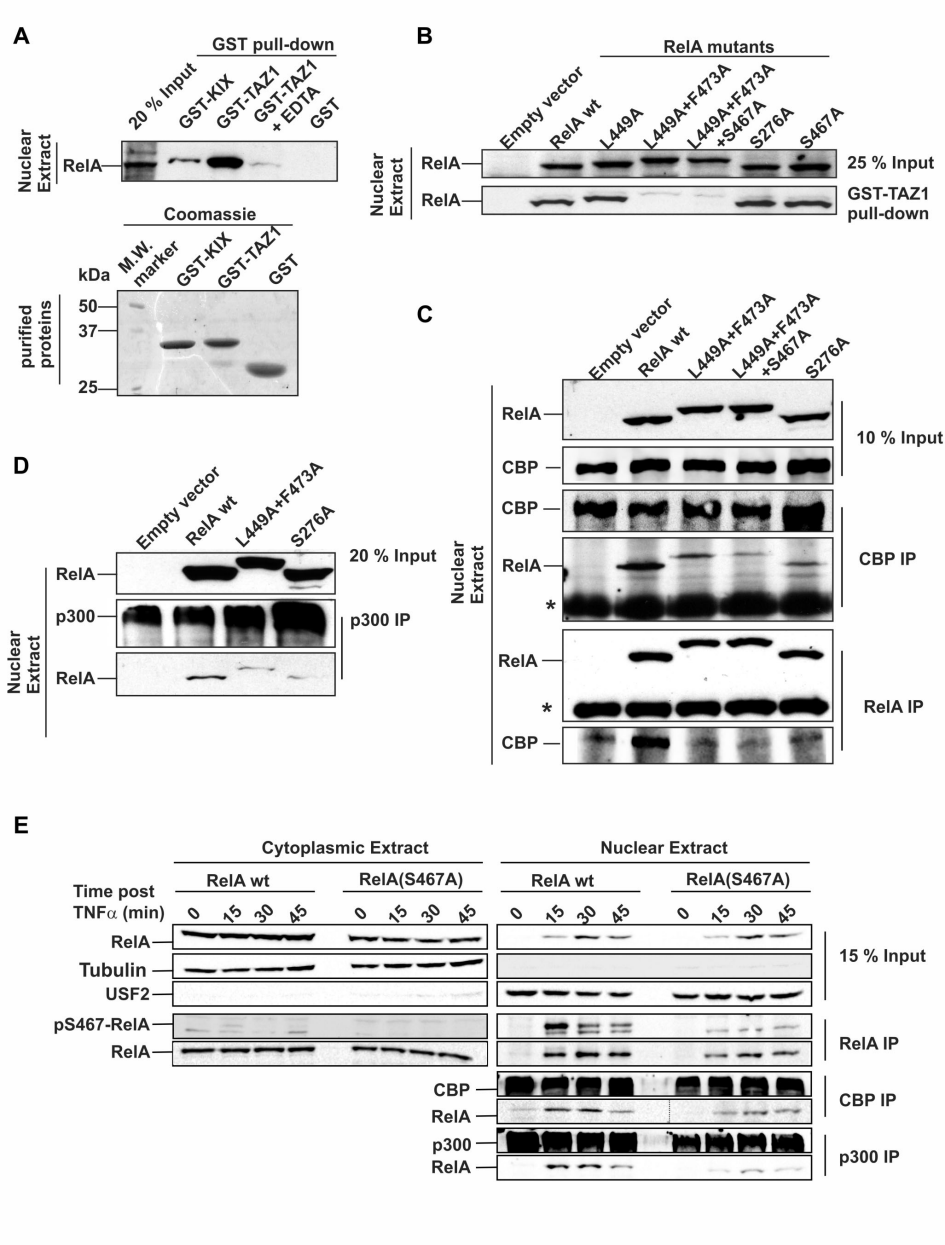
Separating the components of the bipartite RelA:CBP interaction to delineate their individual roles. (A, Upper panel) GST-pulldown assay using in-vitro purified GST–KIX/GST–TAZ1 to pull down nRelA from NEs of wild-type 3T3 cells stimulated with TNFα for 30 min. EDTA was used to remove the Zn^2+^ from TAZ1, thereby disrupting the TAZ1 structure. (Lower panel) Coomassie-stained SDS-PAGE gel showing the inputs for the GST-tagged proteins. (B) GST-pulldown assay using in vitro purified GST–TAZ1 to pull down nRelA from the NEs of RelA(wt/mutants) reconstituted *rela* ^−^/^−^ cells stimulated with TNFα for 30 min. RelA(Leu449Ala+Phe473Ala) and RelA(Leu449Ala+Phe473Ala+Ser467Ala) mutants have slower mobility on SDS-PAGE gels due to unknown reasons. The expression level of the RelA(Ser276Ala) mutant in the reconstituted cell line was lower than that for the RelA(wt) (see Figure S6). Hence, a 3-fold excess of NEs was used for the RelA(Ser276Ala) mutant in this experiment. (C) Interaction of endogenous CBP with nRelA from the NEs of RelA(wt/mutants) reconstituted *rela* ^−^/^−^ cells stimulated with TNFα for 30 min studied by co-immunoprecipitation assay of CBP/RelA followed by immunoblotting by RelA/CBP. The amount of NEs used for RelA(Ser276Ala) mutants was three times that of RelA(wt/TA2) mutants due to its lower expression levels. The RelA(Ser276Ala+Leu449Ala+Phe473Ala) mutant, which could potentially abolish the total RelA:CBP interaction, showed low and inconsistent expression and hence was not used in this study. * denotes IgG heavy chain. (D) The RelA mutants defective in RelA:CBP interaction also are defective binding to p300. The co-IP experiments for p300 was performed similarly to those in panel (C) of this figure. (E) RelA phosphorylated at Ser467 has higher CBP/p300 binding potential than the nonphosphorylated form. The immunoprecipitation assay was performed with α-RelA on CE (left column panels) and NE (right column panels) of *rela* ^−^/^−^ cells reconstituted with RelA(wt) or RelA(Ser467Ala) mutant at three different time intervals after being stimulated with TNFα (5 ng/ml) in addition to the unstimulated cells. Co-immunoprecipitation assays were performed with α-CBP and α-p300 on NE in exactly the same manner as for the IP experiments above. The recruitment of nRelA by CBP/p300 is similar at 15 min relative to 30 min but diminishes at 45 min after TNFα stimulation despite the concentration of total RelA in the nucleus being significantly lower at 15 min after stimulation. Identical co-IP experiments with the RelA(Ser467Ala) mutant shows a direct proportionality in CBP/p300 binding with the concentrations of total nRelA at the different time points after stimulation. This indicates that the exclusively nuclear p-Ser467–RelA whose concentration peaks at about 10 to 15 min post-TNFα stimulation possesses a higher binding affinity for CBP/p300 compared to the nonphosphorylated form.

In addition to the RelA–TA2:TAZ1 interaction, the RelA–RHR interacts with CBP–KIX via p-Ser276–RelA [20]. We compared the binding of RHR to KIX and TA2 to TAZ1 by GST-pulldown assay using nuclear extracts (NEs) of wild-type 3T3 cells stimulated with TNFα for 30 min. KIX and TAZ1 were purified as GST-fusion proteins and used to pull down nuclear-RelA (nRelA). As seen in Figure 4A, significantly lower amounts of nRelA were pulled down by GST–KIX compared to GST–TAZ1, which can be due to either of the two possibilities or both: first, a lower binding affinity of RelA–RHR:KIX compared to RelA–TA2:TAZ1 complex, and second, presence of only a small fraction of p-Ser276–RelA in the total pool of nRelA. In this context, we compared the binding affinities reported in the literature for KIX with other target proteins [32],[33] and found the reported binding affinities for KIX complexes typically 10–100-fold lower than those observed for various TAZ1 complexes [32],[33].

Overall, in either possible scenarios the total amount of RelA–TA2 bound to TAZ1 is significantly higher than that of RelA–RHR bound KIX, which should be reflected in a subset of RelA-activated genes that is regulated through RelA–TA2:TAZ1 interaction and is independent of p-Ser276–RelA:KIX binding.

### RelA–TA2 Mutants Selectively Abrogate the RelA:TAZ1 Component of the Bipartite RelA:CBP/p300 Interaction

Next, we studied the role of the critical hydrophobic residues of RelA–TA2 (Leu449, Leu465, and Phe473) on TAZ1 binding in the context of full-length RelA. We used *in vitro* purified GST-tagged TAZ1 to pull down full-length wild-type (wt) or mutant RelA (Figure 4B) from the NEs of TNFα treated *rela* ^−^/^−^ cells (see Materials and Methods) reconstituted with RelA(wt/mutants). We found that the RelA mutants, namely RelA(Leu449Ala+Phe473Ala) and RelA(Leu449Ala+Phe473Ala+Ser467Ala), were completely defective in binding to TAZ1 (Figure 4B). As expected, the Ser276Ala mutation had no effect on TAZ1 binding. Interestingly, the RelA(Leu449Ala) mutant did not show any noticeable decrease in binding affinity, contrary to the impaired TAZ1 binding observed in our *in vitro* GST pulldown and ITC experiments. A possible explanation is that *in vivo* posttranslational modifications like the phosphorylation of the well-conserved Ser467 could potentially mitigate the effects of the Leu449 mutation, thus increasing binding affinity of the RelA–TA2 to TAZ1, as observed by ITC experiments (Figure 3C and Table 2). Additionally, in the context of full-length RelA, the other domains of RelA could further influence the RelA–TA2:TAZ1 binding.

The above experiments established the minimal subset of mutations required in RelA to selectively prevent its interaction with the TAZ1 domain of CBP. These RelA mutants along with the RelA(Ser276Ala) were further tested for their interaction with endogenous CBP by coimmunoprecipitation (co-IP). Binding of all three RelA mutants [RelA(Leu449Ala+Phe473Ala), RelA(Leu449Ala+Ser467Ala+Phe473Ala), and RelA(Ser276Ala)] to endogenous CBP was impaired relative to RelA(wt) (Figure 4C). However, the disruption of either component of the RelA:CBP interaction—RHR:KIX or TA2:TAZ1—diminished but could not completely abrogate the entire RelA:CBP interaction. The RelA(Leu449Ala+Phe473Ala) and RelA(Ser276Ala) mutants were equally defective in binding to p300 (Figure 4D), whose TAZ1 domains share high sequence identity with CBP [22].

### Role of RelA(Ser467) Phosphorylation in RelA:CBP/p300 Interaction

To further elucidate the role of Ser467 in RelA:CBP/p300 binding, we first studied the RelA(Ser467) phosphorylation event both spatially and temporally following TNFα stimulation. As shown in Figure S6A, the amount of p-Ser467–RelA is negligible in the unstimulated cells. Phosphorylation increases within 5 min of TNFα stimulation, reaching its maximum at 10 to 15 min followed by gradual dephosphorylation starting at 20 min after stimulation. We confirmed the presence of p-Ser467–RelA exclusively in the nucleus [34] (Figure 4E). The timing of RelA nuclear entry coincides with the p-Ser467–RelA maximum, indicating a possible correlation between the Ser467 phosphorylation event and an enhanced early RelA:CBP/p300 interaction. This correlation holds true only if Ser467 phosphorylation contributes towards increasing the affinity. To confirm this, co-IP experiments with α-CBP and α-p300 using NEs of RelA(wt) and RelA(Ser467Ala) mutants at three different time points after TNFα stimulation (Figure 4E) in addition to unstimulated condition were performed. Interestingly, in the RelA(wt) reconstituted cells, despite lower amounts of nRelA at 15 min than at 30 min of TNFα treatment, equivalent amounts of RelA co-immunoprecipitated with CBP/p300. The amount of nRelA bound to CBP/p300 at 45 min after TNFα stimulation diminished significantly. The nonlinearity of the amounts of nRelA co-immunoprecipitated with CBP/p300 with respect to the nRelA concentration is attributed to the enhanced binding affinity of p-Ser467–RelA for CBP/p300, as this nonlinearity was not observed for the identical experiment performed using RelA(Ser467Ala) mutant reconstituted *rela* ^−^/^−^ cells (Figure 4E, Figure S6B).

Despite the role of p-Ser467–RelA in RelA:CBP/p300 binding, RelA(Leu449Ala+Phe473Ala) mutant [henceforth known as the RelA(TA2) mutant] was selected for further gene expression experiments due to two reasons: First, it had the minimal number of mutations required to impair the RelA–TA2:TAZ1 interaction. Second, RelA(Ser467) is a key determining factor for various other processes in the NF-κB pathway [28],[35],[36], which could further complicate and lead to misinterpretation of our experimental observations. Therefore, the RelA(Leu449Ala+Ser467Ala+Phe473Ala) mutant was not used further in this study.

### RelA–TA2:TAZ1 Drives the Transcription of a Subset of NF-κB Target Genes Independent of RelA(Ser276) Phosphorylation

**Figure 5:**
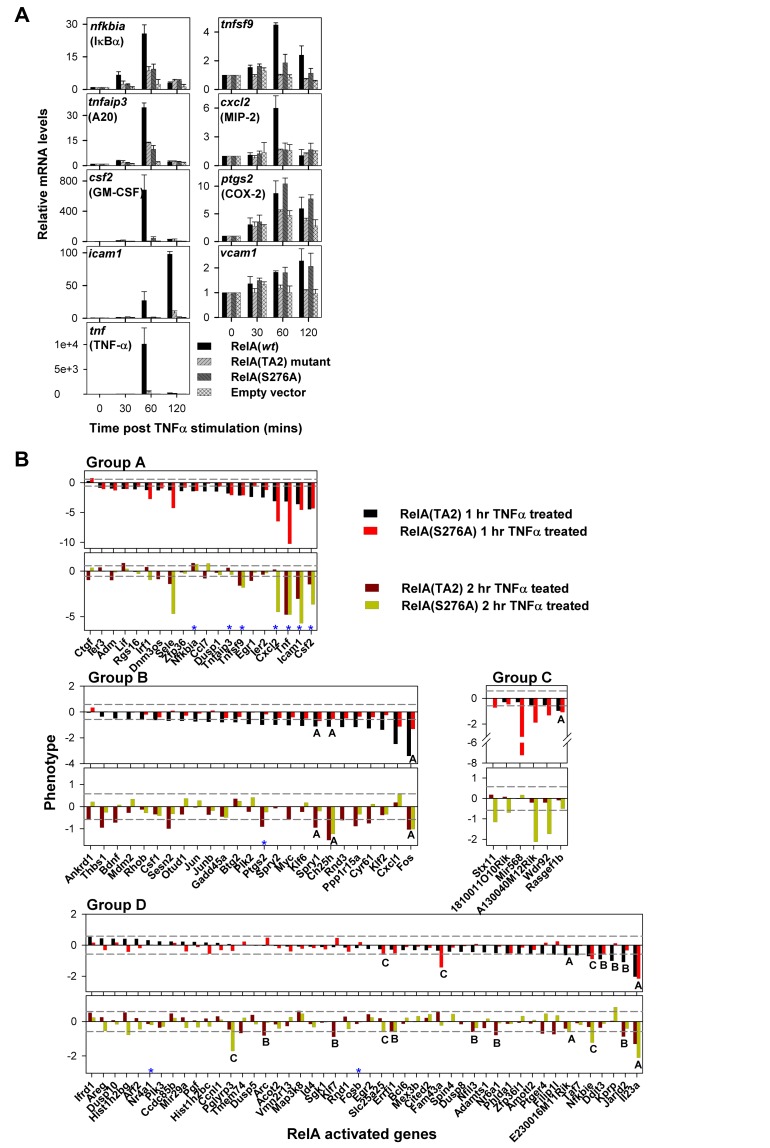
A subset of RelA:CBP/p300 dependent genes is independent of RelA(Ser276) phosphorylation. (A) Total RNA was isolated and purified from unstimulated cells at 30, 60, and 120 min of TNFα stimulation and prepared for analysis by RT-PCR. GAPDH was used as a reference and the 0 min time point for each cell line was used as the calibrator. The respective protein products of the genes (with names other than the gene name) are in parentheses. The *rela* ^−^/^−^ cell line reconstituted with empty vector was included to ensure that RelA regulates the genes under study. The genes with their respective gene accession numbers (mouse) are as follows: *csf2* (NM_009969), *cxcl2* (NM_009140), *icam1* (NM_010493), *nfkbia* (NM_010907), *ptgs2* (NM_011198), *tnf* (NM_013693), *tnfaip3 variant 1* (NM_009397), *tnfaip3 variant 2* (NM_001166402), *tnfsf9* (NM_009404), and *vcam1* (NM_011693). Note that *tnfaip3* has two variants. (B) The RelA–TA2:TAZ1 interaction activates a set of RelA target genes independent of p-Ser276RelA. The expression phenotype was determined for 99 genes that undergo at least 2-fold activation at 1 h of TNFα stimulation as compared to unstimulated RelA(wt) reconstituted *rela* ^−^/^−^ cells. These TNFα-activated genes were grouped into four groups, A–D, based on the observed expression defect (with 95% confidence) with respect to RelA(wt), in the two distinct RelA:CBP/p300 interaction defective mutant cells (see main text). The gene list was sorted according to the differences between the RelA(wt) and RelA(TA2) mutant reconstituted cells at 1 h of TNFα treatment. The genes that switch to other groups upon relaxation of the confidence limit to 67% are labeled with their prospective group names. The genes that were tested by qPCR are marked with a blue asterisk. *vcam1* did not satisfy the stringent criteria of at least 2-fold activation at 1 h of TNFα treatment and hence could not enter the list.

Next we investigated the effect of RelA:CBP/p300 interaction on the inducible expression profile of NF-κB target genes by qRT-PCR. Expression of the target genes was tested in the *rela* ^−^/^−^ cells reconstituted with RelA(wt/mutants) following TNFα stimulation. Fibroblast cells were used because in this cell type the NF-κB-driven transcription is mainly carried out by the RelA subunit [16],[37]. *rela* ^−^/^−^ cells reconstituted with an empty vector were used as a control, which also ensured that the genes tested were RelA dependent. The set of nine RelA-dependent genes are well-studied early TNFα responsive genes (Figure 5A) [16],[19],[38]. In this gene set, *ptgs2* and *nfkbia* are NF-κB target genes believed to be independent of RelA:CBP/p300 interaction [19].

Our qRT-PCR data demonstrated inducible expression for all of the genes in the chosen set following TNFα stimulation in the RelA(wt) reconstituted *rela* ^−^/^−^ cells. However, the expression of all the nine genes was significantly diminished in the cells reconstituted with RelA(TA2) mutant (Figure 5), suggesting that genes like *ptgs2* and *nfkbia*, which are believed to be independent of RelA:CBP/p300 interaction, are actually dependent on RelA:CBP/p300 binding through the RelA–TA2:TAZ1 component (Figure 5A).

On the other hand, for the RelA(Ser276Ala) reconstituted cells, only seven of the nine genes displayed reduced gene expression. The relative mRNA levels of the remaining two genes, namely *vcam1* (NM_011693) and *ptgs2*, were not affected by the RelA(Ser276Ala) mutation following TNFα stimulation (Figure 5). In our study, expression of *nfkbia*, which is believed to be independent of Ser276 phosphorylation, appeared to be partially dependent on it. The lower levels of *nfkbia* mRNA in the RelA(Ser276Ala) reconstituted cell line following TNFα stimulation as compared to those observed for RelA(wt) reconstituted cells could possibly be due to the lower expression of RelA(Ser276Ala) mutant in the cell relative to RelA(wt) and RelA(TA2) mutant (Figure S7).

### Comparative Genome-Wide Expression Analysis Reveals That a Major Set of RelA Target Genes Is Regulated by the RelA–TA2:TAZ1 Interaction

To ascertain the effect of the RelA:CBP/p300 interaction on the RelA-regulated transcriptome, we carried out RNA-seq analysis of total mRNA isolated from RelA(wt/mutants) reconstituted cells prior to TNFα stimulation as well as at two different times points after TNFα stimulation (Figure 5B). The set of genes activated upon TNFα stimulation was classified into four groups: Group A contains genes that are dependent on both the interaction components of the RelA:CBP/p300 complex. Group B contains those dependent only on the RelA–TA2:TAZ1 interaction. Group C contains genes that are dependent only on p-Ser276–RelA, and Group D contains TNFα activated genes, which are RelA regulated but are altogether independent of RelA:CBP/p300 interaction, or the genes that are activated by transcription factors other than RelA. The RNA-seq data revealed that a majority of the RelA:CBP/p300 target genes belong to Group B (25 genes) followed by Group A (20 genes) and Group C (six genes). A sizable number of RelA target genes belong to RelA:CBP/p300 independent Group D (48 genes) (Figure 5B). Based on these experiments, it is clear that the RelA–TA2:TAZ1 interaction regulates a subset of RelA target genes independent of p-Ser276–RelA. Thus, the subset of RelA-activated genes dependent on RelA:CBP/p300 interaction is much larger than previously believed.

The gene expression analysis revealed *nfkbia* (IκBα) and *tnfaip3* (A20), which control the negative feedback loop of the NF-κB pathway, are dependent on RelA:CBP/p300 interaction. IκBα essentially is the main NF-κB inhibitor, which strongly controls the negative feedback and is responsible for the fast turnoff of the NF-κB response after TNFα treatment in fibroblast cells [39]. Therefore, we asked whether reduced expression of *nfkbia* due to defective RelA:CBP/p300 interaction had any effect on the NF-κB pathway.

### RelA:CBP/p300 Interaction Regulates the Temporal Control of IκBα and nRelA

To study the effect of the RelA:CBP/p300 interaction on *nfkbia* expression in detail, we investigated the temporal profile of the IκBα protein in *rela* ^−^/^−^ cells reconstituted with RelA(wt/mutants) following TNFα stimulation. This study was also performed to ensure that the RelA mutations did not interfere with the upstream NF-κB pathway in the cytoplasm or with the DNA binding property of RelA. *nfkbia* is a prominent RelA target gene, which has a robust NF-κB responsive promoter. IκBα is a strong negative regulator of nuclear NF-κB (nNF-κB) and hence a key determinant of the nNF-κB temporal profile [39]. The canonical NF-κB pathway stimulated by TNFα has been extensively studied and focuses on the activation of the IKK-IκB-NF-κB signaling module. A mathematical model, which accounts for experimentally observed nNF-κB activity and IκB expression profiles, has been established [40],[41]. This model depends on the abundance and the transcriptional activation potential of NF-κB (RelA) [42],[43], among other parameters.

**Figure 6:**
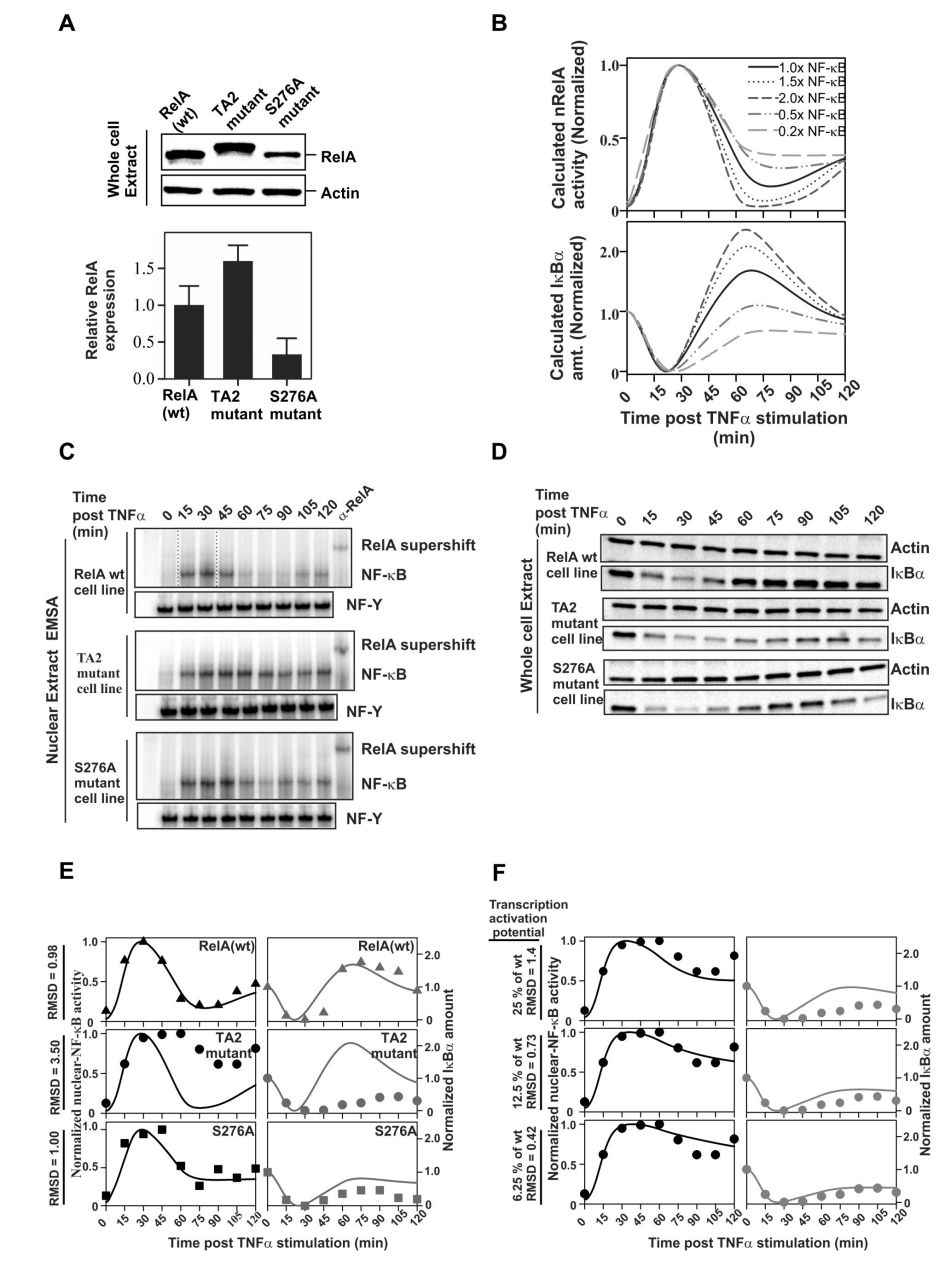
Impaired RelA–TA2:CBP–TAZ1 interaction disrupts the negative feedback loop of the NF-κB pathway. (A) Expression of RelA mutants with respect to RelA(wt) in the RelA reconstituted *rela* ^−^/^−^ cell lines. The protein expression levels of RelA(Ser276Ala) mutant was about three times lower than that for RelA(wt). For the quantitative estimation, the RelA to Actin signal ratio for each cell line was normalized with that for the RelA(wt) reconstituted *rela* ^−^/^−^ cells (lower panel). (B) Model predictions for NF-κB (top) and total IκBα abundance (bottom) for different RelA expression levels (2×, 1.5×, 1×, 0.5×, and 0.2× relative to RelA(wt) values). In the nNF-κB curves, the peak is normalized to one for the highest level of nNF-κB activity and to zero for its levels in the resting cells (time = 0 min) for each individual expression level. Similarly, for the IκBα curves, the amount of IκBα in the resting cell (time = 0 min) is normalized to one and the minimum amount after degradation (basal levels) to zero following TNFα stimulation. (C) nRelA activity assay in response to TNFα stimulation as measured by EMSA using labeled κB probe and control NF-Y probe in the above-mentioned cells. The activity due to RelA binding to the κB probe was indicated by supershift of the EMSA band corresponding to the probe-bound NF-κB detected with anti-RelA antibody. 12 µg of NE was used for EMSA. (D) IκBα degradation and regeneration assay in RelA(wt/mutants) reconstituted *rela* ^−^/^−^ cells following TNFα stimulation. IκBα protein levels after stimulation were monitored with respect to Actin. (E) Comparison of experimentally determined (symbols) and model predictions (solid line) based on adjusted RelA levels for NF-κB (left) and total IκBα abundance (right). Time courses for *rela* ^−^/^−^ cells reconstituted with RelA(wt) (top), RelA(TA2) mutant (middle), and RelA(Ser276Ala) (bottom) stimulated with TNFα are shown. The experimental data were normalized as mentioned in panel (B) of this figure. The RMSD values correspond to the combined NF-κB and IκB datasets. (F) Comparison of experimentally determined (•) and model predictions (solid line) for NF-κB (left column) and total IκBα abundance (right column) for different degrees of suppression of IκBα mRNA production for RelA(TA2) mutant reconstituted *rela* ^−^/^−^ cells.

We observed that the normalized protein levels of RelA in the *rela* ^−^/^−^ cells reconstituted with RelA-mutants varied relative to that in RelA(wt)-reconstituted cells (Figure 6A). This prompted us to simulate the effect of NF-κB abundance on the temporal profile of nNF-κB activity following TNFα stimulation (Figure 6B) before studying the transcriptional activation potential of the RelA-mutants. The simulations show that a decrease in the total NF-κB concentration leads to sustained nNF-κB activity and lower levels of IκBα synthesis following TNFα stimulation (Figure 6B).

We next compared the nuclear translocation of RelA and the temporal profile of IκBα protein following TNFα stimulation in *rela* ^−^/^−^ cells reconstituted with wild-type or mutant RelA. To detect nNF-κB in NEs, the NF-κB DNA binding activity was measured by Electrophoretic Mobility Shift Assay (EMSA) (Figure 6C). nRelA levels were also monitored directly by immunoblotting (Figure S8). The time of entry of RelA(TA2) and RelA(Ser276Ala) mutants into the nucleus after TNFα stimulation is identical to that observed for RelA(wt). However, a prolonged nuclear residence time is observed for RelA(TA2) mutant relative to RelA(wt) reconstituted cells (Figure 6C and Figure S8). We also performed similar time course experiments using the whole cell extracts (WEs) of the respective cells and monitored the IκBα protein levels by immunoblot (Figure 6D). While the time of IκBα degradation and RelA entry into the nucleus are identical in the RelA(wt), RelA(Ser276Ala) and RelA(TA2) mutant reconstituted *rela* ^−^/^−^ cells (Figure 6C,D), the temporal profile of IκBα regeneration and the nuclear residence time of RelA differed significantly (Figure 6C–E). For the RelA(TA2) mutant reconstituted cells, IκBα regeneration is delayed and does not reach its initial levels within the time course of the experiment. The RelA(Ser276Ala) mutant on the other hand did not show any significant delay in IκBα regeneration, although the IκBα level was reduced.

In order to address our observed results, we applied the available mathematical model of NF-κB regulation [40],[41]. We assumed that all parameters other than the RelA protein abundance and its transcriptional activation potential remained unchanged in all of the RelA reconstituted *rela* ^−^/^−^ cell lines. Identical IκBα degradation and nuclear translocation of RelA for the three cell lines validated our assumption (through 30 min after TNFα stimulation in Figure 6C,D and Figure S8). Further, when the cell-line-specific RelA concentration estimates were incorporated into the model, the calculated profile for nNF-κB activity and IκBα abundance matched the experimental data of the RelA(Ser276Ala) mutant to a significant extent (Figure 6E, lowest panel), but did not resemble the observed profiles for the RelA(TA2) mutant (Figure 6E, middle panel). The root mean square deviation (RMSD) was used as a measure of agreement between calculated and the observed data. The results suggest that the observed partial expression of *nfkbia* in RelA(Ser276Ala) mutant reconstituted cells in our qRT-PCR data (Figure 5) was to a great extent due to lower expression of this RelA mutant in the cell line used here.

For the RelA(TA2) mutant, we investigated whether agreement between the experimentally observed and calculated data could be obtained by reducing the NF-κB-dependent IκBα mRNA production rate (k_mRNA_) parameter to 25%, 12.5%, or 6.25% of its value for RelA(wt) (Figure 6F). We found that the lowest k_mRNA_ of 6.25% of RelA(wt) produced the best superimposition of the calculated and experimentally determined temporal profiles of the nNF-κB activity and IκBα regeneration as determined by the RMSD score (Figure 6F, top panel). It should be noted that the k_mRNA_ is directly proportional to the transcriptional activation potential of nNF-κB. Our study suggests that the Leu449Ala and Phe473Ala mutations in RelA lower the transcriptional activation of NF-κB by impairing the RelA–TA2:TAZ1 component of RelA:CBP/p300 interaction.

From the above results we conclude that while *nfkbia* is dependent on RelA–TA2:TAZ1 interaction, it is independent of RelA–RHR:KIX interaction. Thus, our data show that disrupting the RelA–TA2:TAZ1 component of the RelA:CBP/p300 interaction disturbs the temporal profile of the nuclear activity of RelA by interfering with the negative feedback loop of the NF-κB pathway. This could further deregulate the expression of the subset of NF-κB-dependent genes, which are independent of the overall RelA:CBP/p300 interaction.

### RelA:CBP/p300 Interaction at the RelA Target Promoters

**Figure 7:**
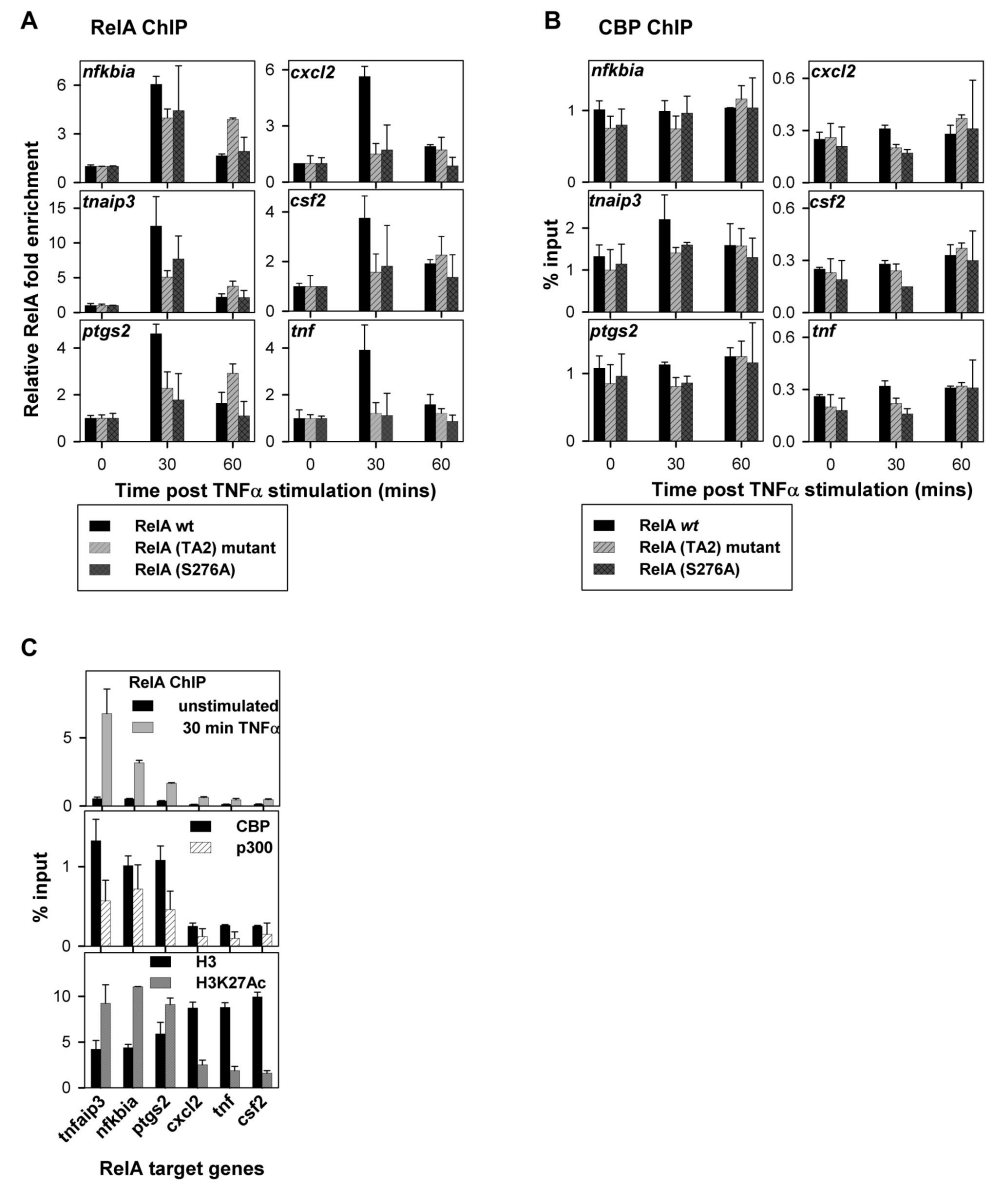
The RelA:CBP/p300 interaction influences RelA recruitment to its target gene promoters. ChIP was performed to analyze the chromatin of six TNFα-induced RelA target genes in unstimulated and TNFα (5 ng/ml) stimulated for 30 and 60 min RelA(wt/mutants) reconstituted *rela* ^−^/^−^ cells. Immunoprecipitation using antibodies against RelA, CBP, p300, histone H3, and H3K27ac were performed. The H3K27ac antibody showed higher signals compared to the total H3 antibody due to differences in their qualities. The results shown are the average of two independent replicates, with standard deviations shown as error bars. (A) Fold enrichment of RelA on the promoter of six RelA target genes after TNFα treatment relative to that in unstimulated cells. (B) CBP/p300 enrichment as depicted by the respective %input for six RelA target genes in unstimulated and TNFα-stimulated RelA(wt/mutants) reconstituted *rela* ^−^/^−^ cells. (C) Comparison of RelA, CBP, p300, H3, and H3K27ac ChIP signals in unstimulated RelA(wt) reconstituted *rela* ^−^/^−^ cells for six RelA target genes.

To further understand the nature of RelA:CBP/p300 interaction on the chromatin, ChIP-qPCR assay was performed to analyze the chromatin at six RelA target genes in unstimulated and TNFα-stimulated RelA(wt/mutants) reconstituted *rela* ^−^/^−^ cells. We examined the chromatin for RelA occupancy on its target promoter set (Figures 7A and S9). As expected, we found that RelA(wt) was recruited to the promoters of all the six genes (although to a varied extent) at 30 min after TNFα treatment. This enrichment of RelA(wt) was diminished at 60 min after stimulation in congruence with its nuclear exit. The recruitment of RelA(TA2) and RelA(Ser276Ala) mutants to the promoter sites of highly expressed *tnfaip3*, *nfkbia*, and *ptgs2* was significantly reduced at 30 min following TNFα treatment compared to RelA(wt), while for the mutants there was no significant RelA signal detected on the promoters with low mRNA expression levels (*cxcl2*, *csf2*, and *tnf*). In agreement with the prolonged nuclear retention of RelA(TA2) mutant due to a defective negative feedback loop, reduced amounts of the RelA(TA2) mutant remained bound to the *tnfaip3*, *nfkbia*, and *ptgs2* promoters even at 60 min after stimulation. As indicated earlier, expression of *tnfaip3* and *nfkbia* were reduced but not completely eliminated. Therefore, the mRNA levels of the RelA target genes correlate well with the amounts of RelA recruited to their respective promoters. These results clearly indicate defects in promoter recruitment of RelA mutants despite their uncompromised DNA binding potential (Figure 6C). This led us to hypothesize that CBP/p300 might be responsible for recruitment of RelA to its target gene promoters. If our hypothesis were true, then CBP/p300 should be constitutively loaded on the promoters and there should exist a direct correlation between the amounts of CBP/p300 present on the promoters to that of RelA recruited upon stimulation. To test our hypothesis, the chromatins were analyzed for CBP/p300 enrichment for our promoter set. The results show CBP/p300 enriched on all of the promoters to a varied extent, with no significant change upon TNFα stimulation (Figures 7B and S9). A comparison of CBP/p300 and RelA signals in this assay revealed a direct correlation between enrichment of RelA upon stimulation with that of CBP/p300 (Figure 7C). Similar RelA recruitment was observed for RelA(wt), RelA(TA2), and RelA(Ser276Ala) mutants on RelA target promoters independent of RelA:CBP/p300 interaction (Figure S10).

Since acetylation of histone-3 (H3) at Lys27 by CBP/p300 is a characteristic of active genes [44], we further examined the levels of H3 occupancy and compared them with that of acetylated histone H3 at Lys 27 (H3K27ac). We expected to observe increased H3K27ac signals on the promoters only after TNFα treatment as a mark of activated promoters as compared to those in unstimulated cells. To our surprise we found H3K27ac enriched in the unstimulated promoters, whose levels remained constant even after TNFα stimulation. Additionally, the H3K27ac levels were directly proportional to the amount of CBP/p300 present on the specific promoters (Figure 7C). This suggests that CBP/p300 bound to promoters of RelA targets is poised for transcription initiation. Upon nuclear entry RelA is recruited to its respective promoter targets by CBP/p300, which is preloaded on these sites, leading to transcription activation.

Upon TNFα stimulation in RelA(TA2) or RelA(Ser276Ala) mutant reconstituted *rela* ^−^/^−^ cells, the mRNA expression levels of the highly expressed genes like *tnfaip3* and *nfkbia* registered only partial reduction (Figure 5A). This is because of a reduced but significant amount of RelA recruited to their respective promoters (Figure 7A). Both the promoters also maintain high levels of CBP/p300, thereby leading to recruitment of the RelA(TA2) and RelA(Ser276Ala) mutants, which possess reduced but significant CBP/p300 binding affinity (Figure 4C,D). This further proves that the RelA-dependent gene expression is regulated by the total amount of RelA recruited on the promoter by CBP/p300.

Overall our study indirectly shows that CBP/p300, which marks the promoter/enhancers of genes, also aids in recruiting RelA to the promoters of its target genes.

## Discussion

In this study, we have provided the structural basis and the functional role of the RelA:CBP/p300 interaction in RelA-driven transcription. In the RelA–TA2:TAZ1 structure, the RelA–TA2 region binds within a series of interlinked hydrophobic grooves that spiral almost entirely around the globular TAZ1 domain. Due to the intrinsically disordered nature of the RelA–TA2 region, binding of an elongated ∼50 amino acid region to TAZ1 in a relatively extended configuration can be accomplished without the energetic cost that would be incurred if unfolding of preexisting globular structure was required. The interaction is mediated primarily by hydrophobic residues interspersed with acidic residues located in the RelA–TA2 region, which dock in the hydrophobic pockets in the surface of TAZ1. While RelA becomes more ordered upon interaction with TAZ1, only limited, highly localized secondary structure is formed. Contacts between the N-terminal part of the RelA–TA2 and TAZ1 are dynamic, contributing little to binding affinity, and are an example of what is frequently termed a “fuzzy” interaction [45]. The hydrophobic contacts are complemented by electrostatic interactions involving the many acidic residues in the RelA–TA2 region. Disruption of any of the hydrophobic contacts leads to a significant decrease in binding affinity toward TAZ1. This is due to the destabilization of the ordered RelA–TA2 structure held primarily by the exposed hydrophobic grooves of TAZ1. Upon disruption of any of the anchor points, RelA–TA2 in absence of any highly stabilized secondary structural elements regains its highly dynamic unstructured high-energy state. This leads to destabilization of the complex and a decrease in TAZ1 binding affinity. Disruption of the hydrophobic anchors of RelA–TA2 can also destabilize the hydrophobic core of TAZ1, which is otherwise stabilized to a significant extent on complex formation with RelA–TA2 (Figure S11). This interlinked core of TAZ1 holds the entire RelA–TA2 region in the complex as seen through the contacts of the core elements with the anchoring residues of RelA–TA2.

The mode of binding of the TAZ1 domain to RelA–TA2 is closely analogous to its interactions with the transactivation domains of the HIF-1α, CITED2, and STAT2 proteins [21]–[24],[46]. In particular, RelA binds in the same hydrophobic grooves as the HIF-1α C-terminal activation domain but in the reverse orientation [21], despite the fact that the two proteins share no common sequence motifs and do not interact with TAZ1 through common secondary structures. A salient property of TAZ1 is its ability to interact with long, negatively charged 40–50 residue regions of intrinsically disordered proteins that dock in the narrow hydrophobic grooves in its surface. This interaction is further enhanced by the phosphorylation of Ser467, thereby adding another layer of regulation for a subset of early response target genes of RelA following TNFα stimulation. Similar phosphorylation events are observed in other transcription factors, which influence their interaction with CBP/p300 [47],[48].

To date, inducible phosphorylation of Ser276 is considered to be the decisive factor for the recruitment of CBP/p300 by RelA for the transcriptional activation of RelA-dependent genes [19],[20]. Earlier studies have shown that in the absence of Ser276 phosphorylation, histone deacetylases (HDACs) could remain bound to nNF-κB and lead to the repression of NF-κB-dependent genes [19],[49]. Upon phosphorylation at Ser276, RelA readily recruits CBP/p300, which in turn acetylates RelA at Lys310, which is further required for the transcription of a subset of RelA-dependent genes [50],[51]. Our experiments show that a major subset of TNFα-inducible genes is dependent on the RelA–TA2:TAZ1 interaction. Importantly, a significant number of these genes (Figure 5B, Group B genes) are activated independently of Ser276 phosphorylation. For these genes the requirement of p-Ser276–RelA could be compensated by a synergy between multiple protein factors (including transcription factors) and RelA. A similar mechanism was previously observed for *cxcl2* and *nfkbia* transcription, where the requirement for RelA:TRAP-80 interaction was waived for the expression of these genes through the recruitment of secondary transcription factors [16]. Similarly, for *tnfaip3* transcription, the constitutive transcription factor Sp1 plays an important role in synergy with RelA [52]. Moreover, the location of Ser276 in the DD of RelA could potentially influence the selectivity of its dimer formation with other NF-κB family members [53]. Additionally, the proximity of Ser276 to DNA binding residues of RelA could further impact the κB site selectivity [54], thereby leading to differential gene expression and/or compensating for the RelA–RHR:KIX interaction.

In this study, for the first time we show that the negative feedback loop is directly controlled by RelA:CBP/p300 interaction through the expression of *nfkbia*. Similarly, A20 protein (*tnfaip3* gene product), another regulator of the negative feedback loop of the NF-κB pathway, is also directly controlled by RelA:CBP/p300 interaction. The impaired negative feedback loop can impact the expression profile of TNFα-induced genes. The longer residence time of RelA in the nucleus can prolong the activation of the genes that are independent of RelA:CBP/p300 interaction.

RelA is believed to recruit co-activator CBP/p300 to the gene promoters for transcription activation of its target genes. On the other hand, recent ChIP-seq and ChIP-chip studies have established p300 as a mark for the enhancers/promoters of active/poised genes throughout the genome including those for RelA target genes [12],[13]. However, the mechanism of its recruitment by RelA to the promoters remains to be established. In this study, we found that CBP/p300 was constitutively enriched on the RelA target promoters as against the TNFα-induced recruitment of RelA to these sites. This points towards a role played by CBP/p300 in recruitment of RelA to its target promoters. Although we have tested only a small set of RelA target promoters, our observation is consistent with the report by Jin et al. [13], where they could accurately predict the target selection by NF-κB in a cell-type-specific manner only when experimentally determined p300 enrichment of promoters was considered along with κB motif [55],[56] along the human genome.

## Materials and Methods

### Protein Expression and Purification

The GST tagged mouse RelA fragments were expressed in *E. coli* [BL21(DE3) cells] and purified by affinity chromatography using Glutathione sepharose beads (GE Life Sciences). The purified protein fragments were then dialyzed in the binding buffer of the respective experiments.

For NMR experiments, the mouse RelA fragments were expressed as fusion proteins with an N-terminal His6-tag followed by the 58 amino acid residue B1 domain of streptococcal protein G (GB1) as per previous reports [57],[58]. The protein was isotopically labeled by growth in M9 minimal media supplemented with ^15^N ammonium chloride and ^13^C-glucose. The fragments were purified under denaturing conditions on a Ni-NTA (SIGMA) column followed by dialysis against cleavage buffer [20 mM Tris (pH 7.5), 200 mM NaCl] at 4°C. The His6–GB1 peptide was cleaved by thrombin at room temperature for 18 h. The cleaved RelA peptides were further purified by reverse-phase HPLC (RPHPLC) using a C18 column on ÄKTA purifier system. The purified peptides were lyophilized immediately. For NMR and ITC experiments, the TAZ1 domain (residues 340–439 of mouse CBP) was expressed and refolded as described previously [24]. For ITC experiments, the His6-tag was cleaved from the GB1–RelA peptides by TEV protease (in house purified).

### GST-Pulldown Assay

The GST-pulldown assay was carried out in binding buffer (20 mM Tris pH 7.5, 150 mM NaCl, and 0.5 mM DTT). GST-RelA fragments (20 µg) were mixed with purified TAZ1 (10 µg) and 20 µl of a 50% slurry of washed glutathione sepharose beads and incubated at 4°C for 30 min in a rotator. The beads were then washed 5 times with 500 µL of binding buffer containing 0.3% TritonX-100, followed by elution and analysis by 15% SDS-PAGE.

### Isothermal Titration Calorimetry (ITC)

ITC experiments were performed at 25°C using a MicroCal Omega VP-ITC instrument. For ITC experiments, GB1-tagged RelA-TA2 fragments were used. The proteins were dialyzed overnight in the ITC buffer [20 mM Tris (pH 8.0), 50 mM or 150 mM NaCl and 1 mM DTT]. The protein concentrations were in the range of 10 to 18 µM of RelA–TA2 peptide in the cell and 169 to 185 µM TAZ1 in the syringe. Protein concentrations were determined by absorbance at 280 nm. The GB1-tag was used for accurate concentration determination of the RelA–TA2 peptides, which have a low extinction coefficient. The GB1-tag did not interact with TAZ1. A typical ITC experiment consisted of a total of 25 injections, using 5–6 µl of TAZ1 for the first injection followed by 24 injections of 11–12 µl of TAZ1 into the cell containing either blank or RelA peptide. Data were analyzed using a single-binding site model in the MicroCal Origin Software. The stoichiometry of binding ranged from 0.9 to 1.2. Errors for the K_d_ values were estimated from duplicate measurements. All the buffers used were made using water purged with nitrogen.

### NMR Spectroscopy and Structure Calculations

NMR experiments were carried out at 25°C on Bruker DRX600 MHz and Avance 900 MHz spectrometers. The typical protein concentration for NMR experiments was 0.7–1.2 mM. All spectra were referenced to external DSS. Refolded TAZ1 and RelA fragments were mixed to form the complex followed by overnight dialysis in NMR buffer (20 mM Tris pH 6.5, 40 mM NaCl, and 2 mM DTT). We added 5% D_2_O to the sample prior to NMR experiments. NMR data were processed using NMRPipe [59] and analyzed using CARA [60]. Backbone and side-chain resonances were assigned using standard triple resonance experiments [61]. Distance restraints were obtained from 3D ^15^N-edited NOESY-HSQC (τ_m_ = 100 ms) and ^13^C-edited NOESY-HSQC (τ_m_ = 100 ms) spectra. Intermolecular NOEs were derived from ^12^C-filtered-^13^C-edited NOESY-HSQC (τ_m_ = 200 ms) [62] experiments. Unambiguous intermolecular NOES were assigned manually and were used in the initial structure calculations, which were performed using CYANA [63] with CANDID [64]. Chemical shift-based torsion angles were obtained using TALOS+ [65]. Distance and torsion angle restraints were imposed to ensure tetrahedral geometry for the zinc atoms of TAZ1 [25]. A total of 200 structures were generated in CYANA and were further refined by restrained molecular dynamics simulated annealing using the AMBER 10 software package [66],[67].

These structures were subjected to 2,000 steps of energy minimization, followed by 20 ps of simulated annealing in vacuum and another 20 ps of simulated annealing using a generalized Born solvent model [68] and finally 2,000 steps of energy minimization. During the simulated annealing, the system was heated to 1,000 K for the first 2 ps, followed by 4 ps at constant temperature, and final cooling to 0 K for the remaining 14 ps. Force constants were 30 kcal mol^−1^ Å^−2^ for NOE restraints and 100 kcal mol^−1^ rad^−2^ for dihedral angle restraints. The 20 lowest energy structures were analyzed using PROCHECK-NMR [69].

### Cell Culture and Reagents

Immortalized *rela* ^−^/^−^ fibroblast cells [70] were reconstituted with retroviral vectors pBABE-puro with mouse RelA(wt/mutants) inserted or with empty vector controls. A final concentration of 5 ng/ml TNFα (Roche Diagnostics) was used for stimulation. Antibodies used for IB and IP in this study were from Santa Cruz: anti-RelA (sc-372g), anti-IκBα (sc-371), anti-CBP (sc-369), anti-Actin (sc-1615) and anti-USF2 (sc-861), anti-α-Tubulin (sc-5286), anti-p300 (sc-584 and sc-585), and Cell-Signaling Technology: p-Ser468-RelA (#3039). cOmplete, EDTA-free Protease Inhibitor cocktail was from Roche Diagnostics. Protein G-beads were from GE Life Sciences.

### Preparation of Cytoplasmic, Nuclear, and WE

For the NE, 3T3 cells were cultured in 10 cm^3^ plates and harvested using PBS buffer. Cell pellets were prepared by 5 min centrifugation at 500 *g* at 4°C and then resuspended in CE buffer (0.32 M Sucrose, 10 mM Tris HCl pH 8.0, 3 mM CaCl_2_, 2 mM MgOAc, 0.5% NP-40, 1 mM DTT, 0.5 mM PMSF) followed by centrifugation at 500 *g* for 5 min at 4°C. The supernatant (CE) was flash frozen and stored at −80°C. The nuclear pellet was washed twice with CE buffer without NP-40 followed by centrifugation as above. The washed pellet was resuspended in hypotonic buffer [20 mM HEPES (pH 7.9), 1.5 mM MgCl_2_, 20 mM KCl, 25% glycerol, 0.5 mM DTT, 0.5 mM PMSF] followed by gradual addition of high salt buffer [20 mM HEPES (pH 7.9), 1.5 mM MgCl_2_, 800 mM KCl, 25% glycerol, 1% NP-40, 0.5 mM DTT, 0.5 mM PMSF, and 1× complete EDTA-free protease inhibitor]. The samples were incubated with mild agitation for 45 min at 4°C followed by centrifugation at 12,000 *g* at 4°C for 15 min. The supernatant was collected as NE and was further subjected to quantification.

For WE, the cells were harvested as above followed by addition of RIPA lysis buffer containing protease inhibitor cocktail. The samples were centrifuged at 12,000 *g* at 4°C. The supernatant was collected as the WE. All of the buffers contained phosphatase inhibitor cocktail.

### Immunoblotting and Co-Immunoprecipitation

For immunoblotting, WEs and NEs were resolved on SDS-PAGE and immunoblotted with respective antibodies. For co-immunoprecipitation (co-IP) assay, NEs were incubated with anti-RelA or anti-CBP antibodies for 1 h followed by addition of prewashed protein-G beads and incubation for 4 h. The beads were then washed thoroughly and resolved on SDS-PAGE and immunoblotted with anti-RelA and anti-CBP antibodies. For quantitative analysis of the immunoblots, the loading controls were analyzed from the same gel as for the protein monitored.

### Gene Expression Analysis

RNA was extracted and purified as per the manufacturer's protocol using the RNeasy kit (QIAGEN). We reverse transcribed 1 µg of the quantified RNA using superscript II RT system (Invitrogen) and poly-dT primers. cDNA fragments generated were analyzed by qPCR using the KAPA system and Realplex thermocycler (EPPENDORF). PCR amplification conditions were 95°C (4 min) and 40 cycles of 95°C (15 s), 56°C (30 s), and 72°C (30 s). Primer pairs used were from previous studies [38],[71] and were tested by melt-curve analysis. Δ(ΔCt) method was used for data analysis with *gapdh* as normalization control to derive fold induction over basal levels. For each set of time-course experiment (0 min, 10 min, 60 min, and 120 min) RNA was collected from empty vector, RelA(wt), RelA(TA2), RelA(Ser276Ala) mutant reconstituted *rela* ^−^/^−^ cells stimulated with 5 ng/ml TNFα. For qRT-PCR experiments, the data were collected for three set of biological replicates.

Next generation sequencing was performed at the UCSD Biogem core facility. Reads were obtained from Illumina HiSeq 2000 sequencing system. Sequencing experiments were performed on the total mRNA extract from RelA(wt), RelA(TA2), and RelA(Ser276Ala) reconstituted rela^−^/^−^ cells at two different time points after TNFα (5 ng/ml) treatment in addition to unstimulated cells. RNA-seq was performed on nine different experimental conditions in total. Two independent replicates were used for each experimental condition. Reads were mapped to the mm10 mouse genome using TopHat [72], and the transcriptome assembly was generated using Cufflinks [73] followed by detection of differentially expressed genes using Cuffdiff.

### Computational Simulation

The ordinary differential equation (ODE)-based model for NF-κB regulation [40] was adapted (Table S1) by increasing IκB mRNA degradation rates by a factor of 1.8 in line with recent measurements [74] and replacing the terms describing transcription-factor-dependent mRNA production for IκBα and IκBε by those used in [41] (Model 3, additive Pol II recruitment). Model simulations were carried out with the “Stiffness Switching” method of the NDSolve function in the package Mathematica 8 (Wolfram Research, Champagne, IL) using a numerically defined IKK activity induced by exposure to TNFα Table S2 [40].

### ChIP Experiments

Samples for ChIP experiments were prepared using SimpleChIP Plus Enzymatic chromatin IP kit (Cell Signaling Technology #9005) according to the manufacturer's protocol with some modifications. Primer pairs used were from previous studies [38],[71],[75] and were tested by melt-curve analysis. ChIP experiments were performed with anti-H3 (Cell Signaling #4620), anti-acetyl H3K27 (Cell Signaling #8173), anti-RNA Pol II (Santa Cruz sc-900), anti-p300 (Santa Cruz sc-585), anti-CBP (Santa Cruz sc-369), anti-RelA (Santa Cruz sc-372), and anti-IgG (Cell Signaling #2729)

### Accession Numbers

Coordinates and structural restraints for the RelA–TA2:CBP–TAZ1 complex have been deposited in the Protein Data Bank under the accession number 2LWW. The chemical shifts have been deposited in the BioMagResBank, accession number 18650. The RNA-seq data discussed in this publication have been deposited in NCBI's Gene Expression omnibus [76] and are accessible through GEO Series accession number GSE46213. (http://www.ncbi.nlm.nih.gov/geo/query/acc.cgi?acc=GSE46213).

## Supporting Information

Figure S1 **Optimization of RelA–TAD fragment in RelA–TAD:TAZ1 complex for structural analysis.** (A) Schematic of GST-tagged RelA–TAD constructs. All constructs were overexpressed and purified for the GST-pulldown assays. Construct T11 (marked with an asterisk) was determined to be the optimal fragment for CBP–TAZ1 binding. (B) GST-pulldown experiment for optimization of the N-terminal end of the RelA fragment binding to TAZ1. (C) GST-pulldown experiment for optimization of the C-terminal end of the RelA fragment binding to TAZ1. (D) GST-pulldown experiment for studying the importance of the five amino acids at the C-terminal end of the RelA fragment binding to TAZ1. (E) Gel filtration elution profile of CBP–TAZ1 domain, RelA–TA2 fragment, and RelA–TA2:CBP–TAZ1 complex. The RelA–TA2 fragment was detected at 215 nm due to lack of tyrosine and tryptophan residues. RelA–TA2:TAZ1 complex and free TAZ1 were detected at 280 nm. A slight excess of TAZ1 was used for the complex formation as can be seen in the elution profile of the complex. (F) Gel filtration elution characteristics of CBP–TAZ1 domain, RelA–TA2 fragment, and RelA–TA2:CBP–TAZ1 complex. The observed molecular weight of RelA–TA2 fragment is about 3 times that of its calculated molecular weight, which further confirms the unstructured nature of RelA–TA2 concluded from its [^15^N-^1^H]-HSQC spectra in Figure 1C. Zn^2+^-bound well-folded TAZ1 elution profile corresponds to that of a lower calculated molecular weight protein.
(TIF)

Figure S2 **TAZ1 binds to RelA–TA2 through a series of interlinked hydrophobic grooves.** (A) Cartoon representation of the hydrophobic core of TAZ1. The critical amino acid residues of the core are shown as pink sticks. These core residues pack the α_1_, α_2_, and α_3_ helices of TAZ1. (B) Surface representation of TAZ1 (in the RelA–TA2:TAZ1 complex). The hydrophobic core residues are colored in pink, and additional hydrophobic residues, which contribute to each of the grooves, are shown in blue. The three hydrophobic groves are interlinked with each other through the hydrophobic residues of the core (pink). Figure S2A and the left panel in Figure S2B are represented in the same orientation.
(TIF)

Figure S3 **The α_N_ helix of RelA–TA2 in the RelA–TA2:TAZ1 complex is dynamically disordered.** (A) Histograms of [^1^H]-^15^N heteronuclear NOE values for ^15^N-labeled RelA–TA2 in complex with unlabeled TAZ1 (top panel) and ^15^N-labeled TAZ1 in complex with unlabeled RelA–TA2 (bottom panel). The secondary structural elements are represented schematically above the corresponding plots. Higher values of NOEs correspond to more rigid structure. (B) Chemical shift deviation (CSD) of Cα (top panel) and C′ (bottom panel) for RelA–TA2 in the complex from that of their sequence-corrected random coil chemical shifts. The percentage of the helix population for the RelA–TA2 region in the complex was estimated from the CSDs of Cα and C′ chemical shifts averaged from Leu434–Leu439 for the α_N_ and from Ser471–Leu477 for the α_C_ helix. The secondary chemical shift for a fully formed α-helix is 2.8 ppm and 2.1 ppm for Cα and C′, respectively [26]. The estimated helical population from the average CSD for the α_N_ and α_C_ terminal helix of RelA–TA2 is 32% and 84%, respectively.
(TIF)

Figure S4 **RelA–TA2 docks into the grooves formed by the opposite faces of the hydrophobic core of TAZ1.** (A) A surface representation of TAZ1 in the RelA–TA2:TAZ1 complex. The hydrophobic core residues of TAZ1 are in pink, and the additional residues of TAZ1 that contribute towards RelA–TA2 binding are colored blue. RelA–TA2 is shown as grey ribbon with its anchoring hydrophobic residues depicted as green ball-and-sticks. For RelA–TA2, only the residues with more than 10 intermolecular NOEs are depicted as ball-and-sticks. (B) Histograms showing the number of intermolecular NOEs between RelA–TA2 and TAZ1 observed per residue of RelA–TA2 in the complex. Compared to the α_C_ helix, the α_N_ helix of RelA–TA2 has fewer intermolecular NOEs as expected from its dynamic nature (see Figure S3).
(TIF)

Figure S5 **ITC binding isotherms for the RelA(TA2) (wt/mutants) with TAZ1.** The TAZ1 binding affinity is completely abolished in the RelA(TA2) (Phe473Ala) mutant. The RelA–TA2 fragment of Lys425–Arg508 was used for all studies (wt and mutants) unless otherwise mentioned.
(TIF)

Figure S6 (A) The RelA(Ser467) phosphorylation maximum coincides with nuclear entry of RelA after TNFα stimulation. (A) RelA immunoprecipitation assay was used to detect RelA(Ser467) phosphorylation in WEs of RelA(wt) and RelA(Ser467Ala) reconstituted *rela* ^−^/^−^ cells following stimulation with 5 ng/ml TNFα for the indicated time interval. 150 µg of WE was used for IP. (B) RelA(Ser467) phosphorylation enhances the binding affinity of RelA for CBP/p300. (Top panel) Nuclear translocation assay in RelA(wt) (black) and RelA(Ser467Ala) (red) mutant reconstituted *rela* ^−^/^−^ cells following stimulation with 5 ng/ml TNFα (data from Figure 4E). The amount of p-Ser467–RelA (grey) was monitored by RelA IP of NE at mentioned time intervals after TNFα treatment. (Bottom panel) The RelA:CBP/p300 interaction at various time intervals after TNFα stimulation was monitored by Co-IP experiments using CBP/p300 antibodies for RelA(wt) (black) and RelA(Ser467Ala) mutant (red) (from Figure 4E). The binding curve for RelA(wt) is shifted to the left, where the concentration of p-Ser467–RelA is highest. The RelA(Ser467Ala) mutant shows a linear change in the aforementioned binding with respect to the concentration of total nRelA. The lines joining the data points are only used for the purpose of viewing clarity.
(TIF)

Figure S7 **Expression levels of RelA in the RelA reconstituted** ***rela*** **^−^/^−^ cells.** RelA protein levels (wt and mutants) were determined in WEs of the RelA reconstituted *rela* ^−^/^−^ cell lines detected by immunoblotting of RelA. Actin was used as the loading control.
(TIF)

Figure S8 **The nuclear residence time of the RelA(TA2) mutant is longer than RelA(wt) and RelA(Ser276Ala).** RelA nuclear translocation assay in RelA(wt/mutants) reconstituted *rela* ^−^/^−^ cells following stimulation with 5 ng/ml TNFα. Upstream stimulatory factor 2 (USF2) was used as the loading control for the NEs.
(TIF)

Figure S9 **CBP/p300 aids recruitment of RelA to its target promoters.** ChIP experiments were performed with RelA, CBP, p300, H3, H3K27ac, RNA polymerase II, and IgG antibodies in RelA(wt), RelA(TA2), or RelA(Ser276Ala) reconstituted *rela* ^−^/^−^ cells under unstimulated or stimulated conditions for 30 min or 60 min with TNFα. Results are presented as average %input values with standard deviations from two independent chromatin preparations. The respective gene names are mentioned on the *y*-axis of each group of panels.
(TIF)

Figure S10 **RelA(TA2) mutant has normal promoter recruitment for RelA:CBP/p300 interaction-independent RelA-regulated genes.** (A) Gene expression profile of *fosb* and *nr4a1* genes. *fosb* (NM_008036) and *nr4a1* (NM_010444) genes belong to the Group D genes (Figure 5B). Kasper et al. have shown both *nr4a1* and *fosb* were highly expressed following forskolin stimulation in *cbp* ^−^/^−^ and *p300* ^−^/^−^ MEF cells [75], thus indicating their CBP/p300-independent expression. Here both the genes showed higher TNFα-induced expression in empty vector reconstituted *rela* ^−^/^−^ cells compared to the RelA(wt/mutant) reconstituted cells. The level of TNFα-induced expression of *nr4a1* and *fosb* was reduced in the presence of RelA(wt) as well as mutants to a similar extent. This suggests that the transcription of both the genes is regulated by RelA but is independent of RelA:CBP/p300 interaction. (B) RelA(wt), RelA(TA2) mutant, and RelA(S276) mutant are recruited to a similar extent on the promoters of *fosb* and *nr4a1*. ChIP-qPCR experiments were performed as mentioned earlier (Figure 7) to study RelA recruitment on the promoter sites of *fosb* and *nr4a1*.
(TIF)

Figure S11 **The hydrophobic core of TAZ1 is stabilized upon binding to RelA–TA2 as seen from the deuterium exchange experiments of ^15^N-labeled TAZ1 and ^15^N-labeled TAZ1 in complex with unlabeled RelA–TA2.** (A, Top row) 2D [^15^N–^1^H]-HSQC spectrum of free TAZ1 in H_2_O (left panel), 11 min after addition of D_2_O (middle panel), and about 33 min (right panel) after addition of D_2_O. (Bottom row) 2D [^15^N–^1^H]-HSQC spectrum of TAZ1 in complex with unlabeled RelA–TA2 in H_2_O (left panel), 11 min after addition of D_2_O (middle panel), and about 21 h (right panel) after addition of D_2_O. The time reported here is of the first free induction decay (fid) recorded after addition of D_2_O. (B) Cartoon representations of TAZ1 with amino acid residues observed in the 2D [^15^N–^1^H]-HSQC spectrum post-D_2_O exchange with higher resistance to exchange are shown as blue sticks. The residues with higher resistance toward D_2_O exchange in TAZ1 (in complex with RelA–TA2) belong either to the hydrophobic core (Leu357, Leu391, and Ile415) or around it (Leu360, Gln355, Val358, Leu359, and Leu361).
(TIF)

Table S1 **Parameters for the ODE-based model for NF-κB regulation.**
(DOC)

Table S2 **Changes in IKK activity induced by exposure to TNFα.**
(DOC)

Text S1 **Supporting procedures legends, deuterium exchange of backbone amide protons, and gel filtration.**
(DOC)

## Abbreviations

CBP: CREB binding protein
GST: glutathione S-transferase
NF-κB: nuclear factor kappa B
nRelA: nuclear-RelA
TNFα: tumor necrosis factor alpha
