# Supplementary figures and images for: Analysis of the RelA:CBP/p300 Interaction Reveals Its Involvement in NF-κB-Driven Transcription
Source: PLoS Biol. 2013 Sep 3;11(9):e1001647. doi: 10.1371/journal.pbio.1001647 (PMC3760798; doi:10.1371/journal.pbio.1001647)

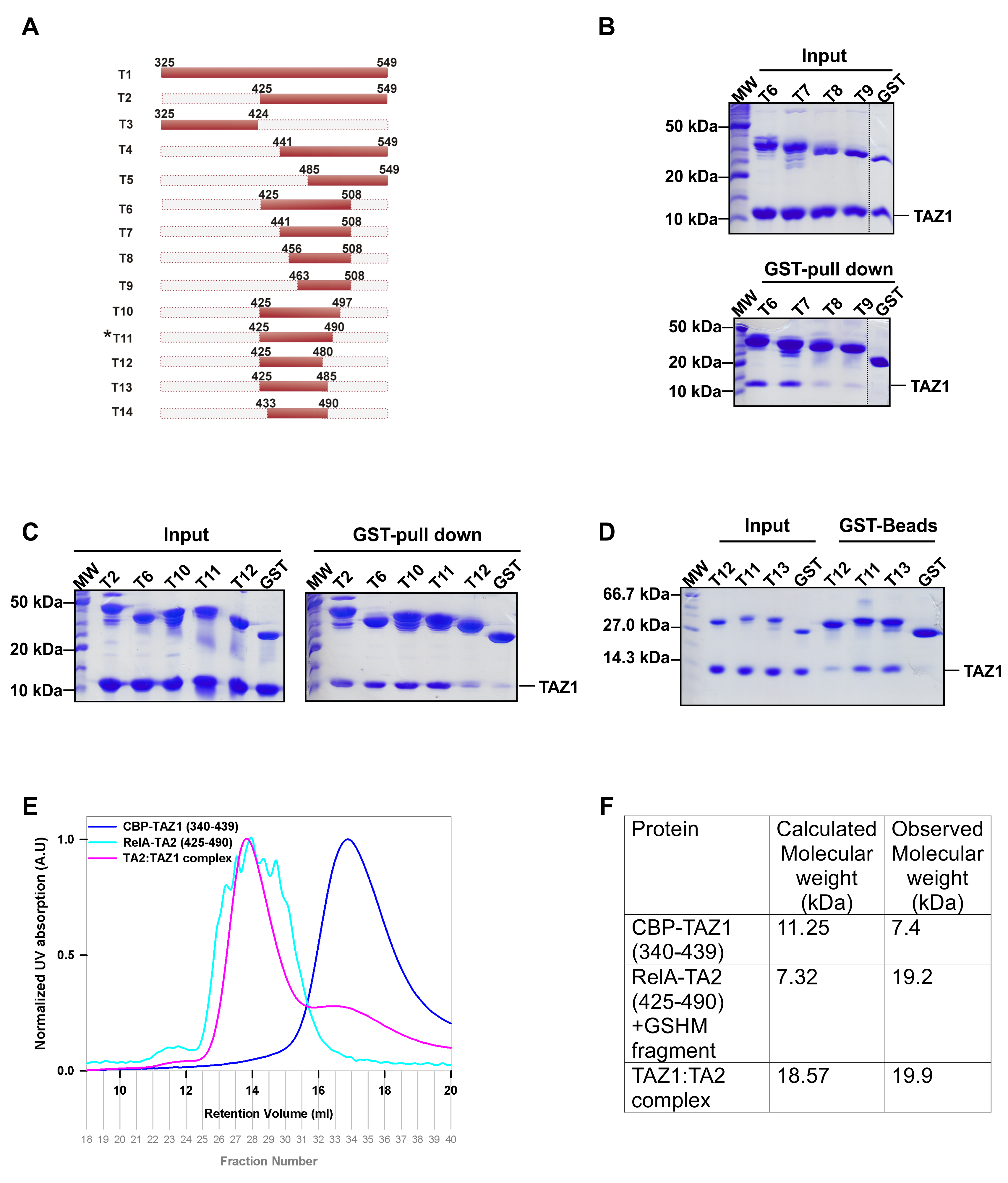

Supplement: Figure S1 — Optimization of RelA–TAD fragment in RelA–TAD:TAZ1 complex for structural analysis. (A) Schematic of GST-tagged RelA–TAD constructs. All constructs were overexpressed and purified for the GST-pulldown assays. Construct T11 (marked with an asterisk) was determined to be the optimal fragment for CBP–TAZ1 binding. (B) GST-pulldown experiment for optimization of the N-terminal end of the RelA fragment binding to TAZ1. (C) GST-pulldown experiment for optimization of the C-terminal end of the RelA fragment binding to TAZ1. (D) GST-pulldown experiment for studying the importance of the five amino acids at the C-terminal end of the RelA fragment binding to TAZ1. (E) Gel filtration elution profile of CBP–TAZ1 domain, RelA–TA2 fragment, and RelA–TA2:CBP–TAZ1 complex. The RelA–TA2 fragment was detected at 215 nm due to lack of tyrosine and tryptophan residues. RelA–TA2:TAZ1 complex and free TAZ1 were detected at 280 nm. A slight excess of TAZ1 was used for the complex formation as can be seen in the elution profile of the complex. (F) Gel filtration elution characteristics of CBP–TAZ1 domain, RelA–TA2 fragment, and RelA–TA2:CBP–TAZ1 complex. The observed molecular weight of RelA–TA2 fragment is about 3 times that of its calculated molecular weight, which further confirms the unstructured nature of RelA–TA2 concluded from its [15N-1H]-HSQC spectra in Figure 1C. Zn2+-bound well-folded TAZ1 elution profile corresponds to that of a lower calculated molecular weight protein. (TIF) [file pbio.1001647.s001.tif]

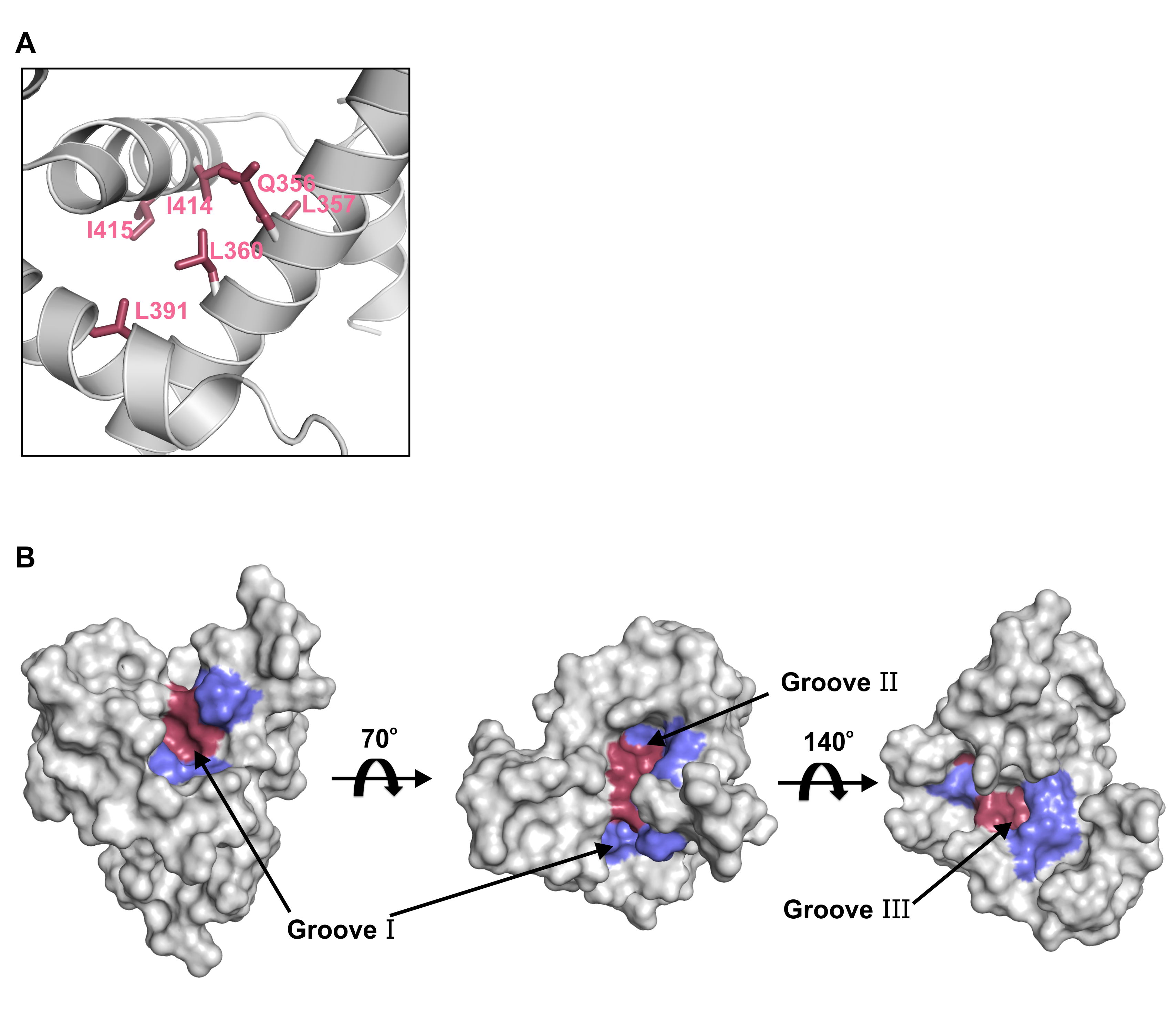

Supplement: Figure S2 — TAZ1 binds to RelA–TA2 through a series of interlinked hydrophobic grooves. (A) Cartoon representation of the hydrophobic core of TAZ1. The critical amino acid residues of the core are shown as pink sticks. These core residues pack the α1, α2, and α3 helices of TAZ1. (B) Surface representation of TAZ1 (in the RelA–TA2:TAZ1 complex). The hydrophobic core residues are colored in pink, and additional hydrophobic residues, which contribute to each of the grooves, are shown in blue. The three hydrophobic groves are interlinked with each other through the hydrophobic residues of the core (pink). Figure S2A and the left panel in Figure S2B are represented in the same orientation. (TIF) [file pbio.1001647.s002.tif]

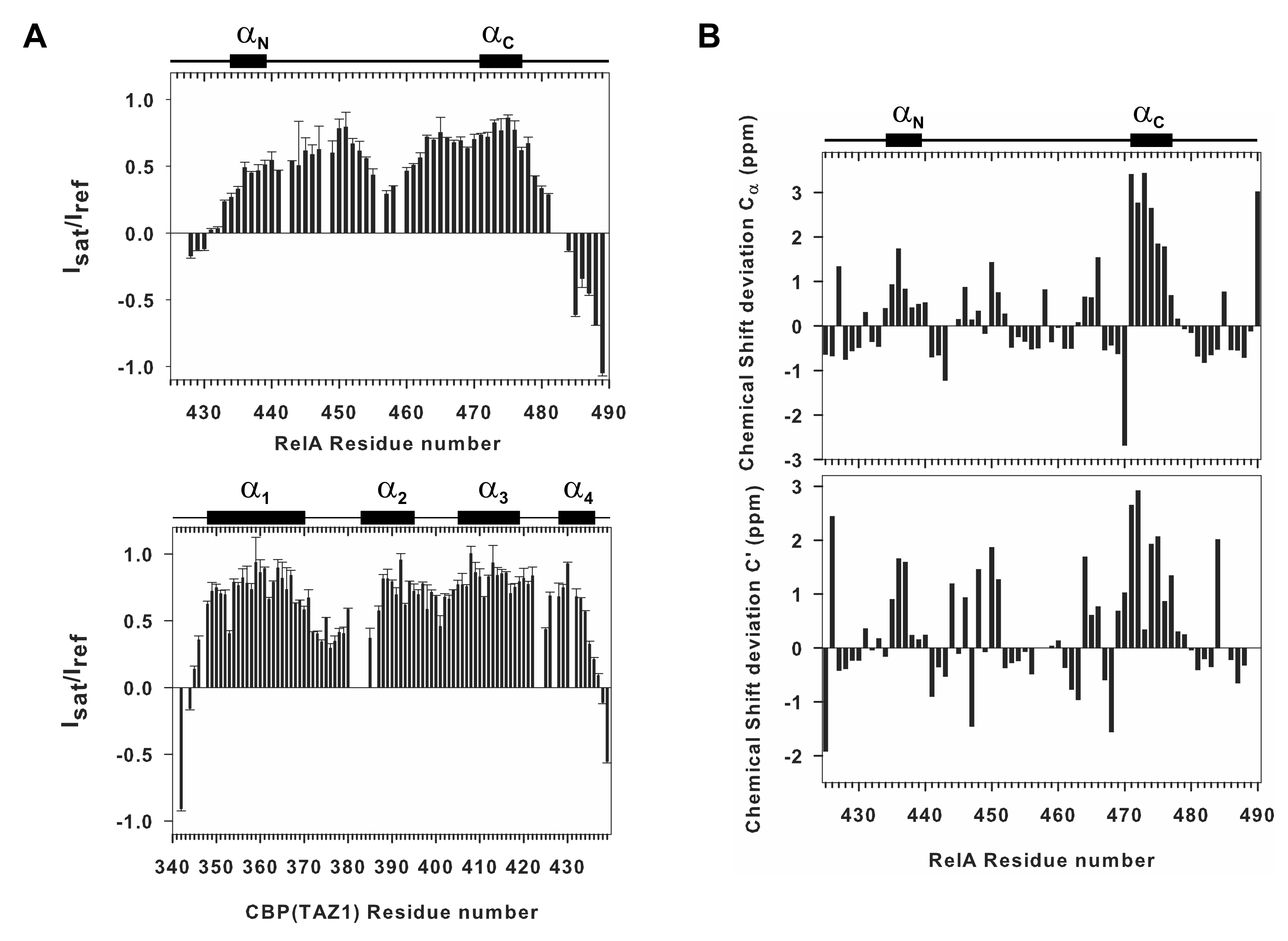

Supplement: Figure S3 — The αN helix of RelA–TA2 in the RelA–TA2:TAZ1 complex is dynamically disordered. (A) Histograms of [1H]-15N heteronuclear NOE values for 15N-labeled RelA–TA2 in complex with unlabeled TAZ1 (top panel) and 15N-labeled TAZ1 in complex with unlabeled RelA–TA2 (bottom panel). The secondary structural elements are represented schematically above the corresponding plots. Higher values of NOEs correspond to more rigid structure. (B) Chemical shift deviation (CSD) of Cα (top panel) and C′ (bottom panel) for RelA–TA2 in the complex from that of their sequence-corrected random coil chemical shifts. The percentage of the helix population for the RelA–TA2 region in the complex was estimated from the CSDs of Cα and C′ chemical shifts averaged from Leu434–Leu439 for the αN and from Ser471–Leu477 for the αC helix. The secondary chemical shift for a fully formed α-helix is 2.8 ppm and 2.1 ppm for Cα and C′, respectively [26]. The estimated helical population from the average CSD for the αN and αC terminal helix of RelA–TA2 is 32% and 84%, respectively. (TIF) [file pbio.1001647.s003.tif]

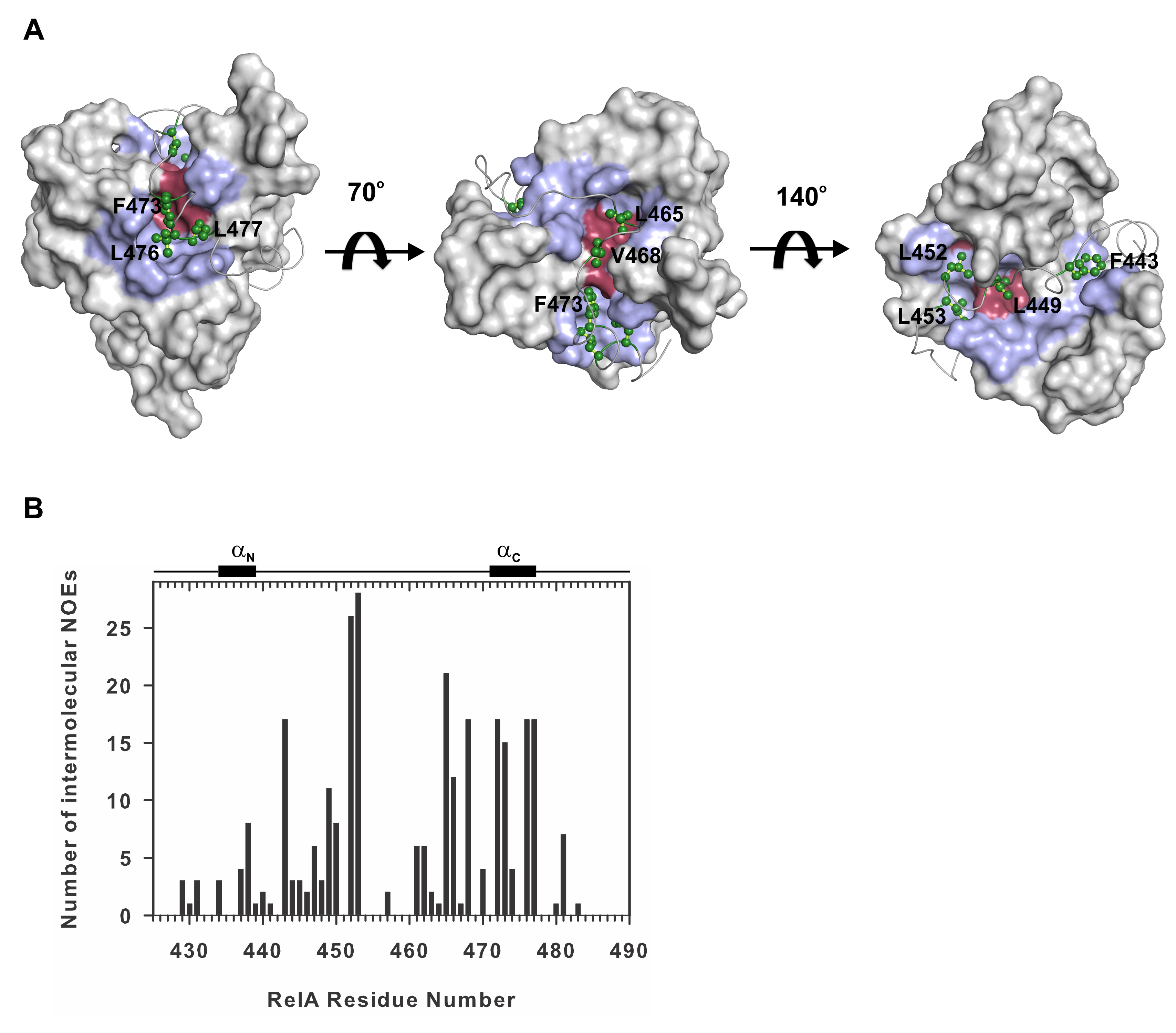

Supplement: Figure S4 — RelA–TA2 docks into the grooves formed by the opposite faces of the hydrophobic core of TAZ1. (A) A surface representation of TAZ1 in the RelA–TA2:TAZ1 complex. The hydrophobic core residues of TAZ1 are in pink, and the additional residues of TAZ1 that contribute towards RelA–TA2 binding are colored blue. RelA–TA2 is shown as grey ribbon with its anchoring hydrophobic residues depicted as green ball-and-sticks. For RelA–TA2, only the residues with more than 10 intermolecular NOEs are depicted as ball-and-sticks. (B) Histograms showing the number of intermolecular NOEs between RelA–TA2 and TAZ1 observed per residue of RelA–TA2 in the complex. Compared to the αC helix, the αN helix of RelA–TA2 has fewer intermolecular NOEs as expected from its dynamic nature (see Figure S3). (TIF) [file pbio.1001647.s004.tif]

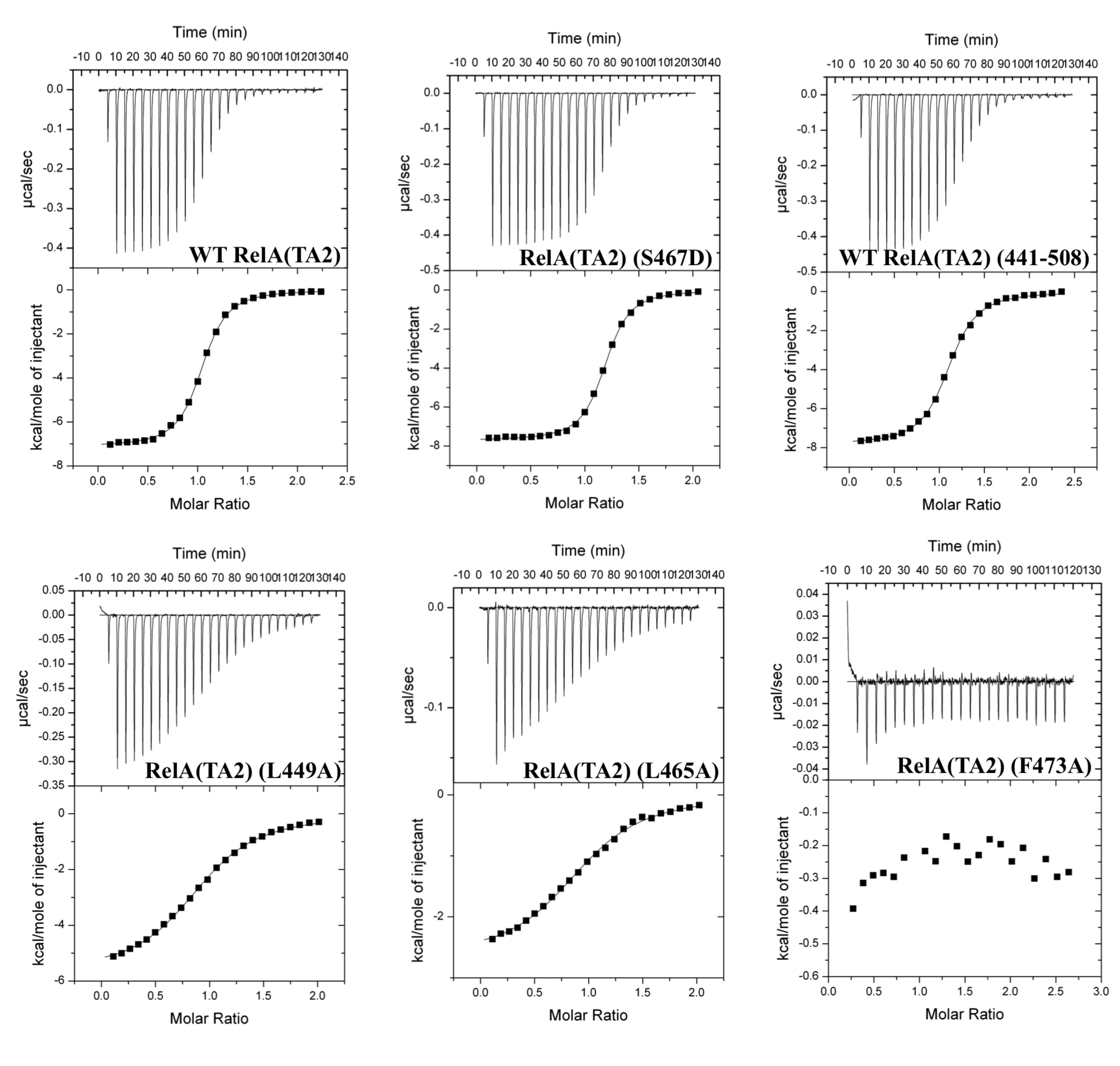

Supplement: Figure S5 — ITC binding isotherms for the RelA(TA2) (wt/mutants) with TAZ1. The TAZ1 binding affinity is completely abolished in the RelA(TA2) (Phe473Ala) mutant. The RelA–TA2 fragment of Lys425–Arg508 was used for all studies (wt and mutants) unless otherwise mentioned. (TIF) [file pbio.1001647.s005.tif]

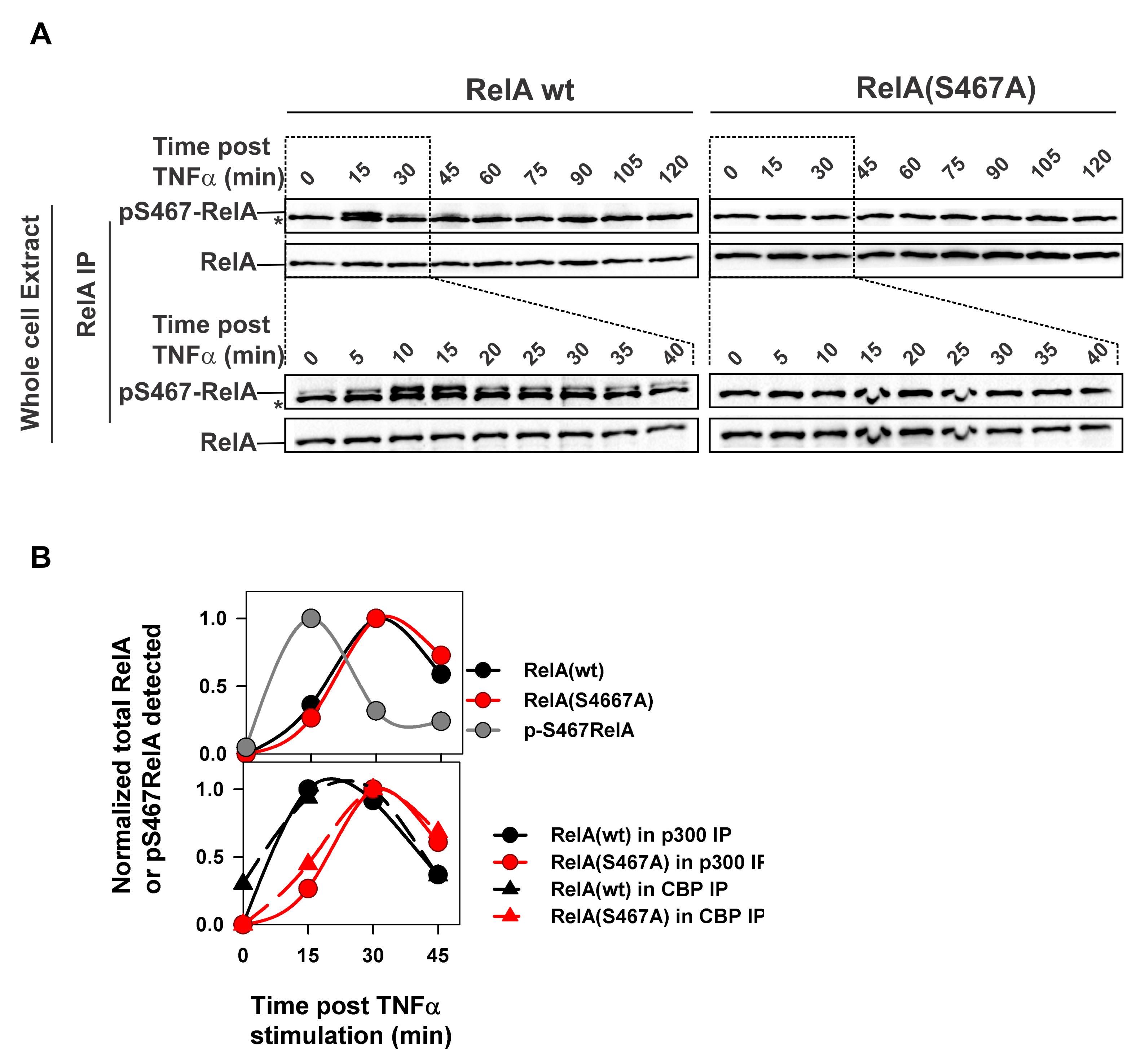

Supplement: Figure S6 — (A) The RelA(Ser467) phosphorylation maximum coincides with nuclear entry of RelA after TNFα stimulation. (A) RelA immunoprecipitation assay was used to detect RelA(Ser467) phosphorylation in WEs of RelA(wt) and RelA(Ser467Ala) reconstituted rela −/− cells following stimulation with 5 ng/ml TNFα for the indicated time interval. 150 µg of WE was used for IP. (B) RelA(Ser467) phosphorylation enhances the binding affinity of RelA for CBP/p300. (Top panel) Nuclear translocation assay in RelA(wt) (black) and RelA(Ser467Ala) (red) mutant reconstituted rela −/− cells following stimulation with 5 ng/ml TNFα (data from Figure 4E). The amount of p-Ser467–RelA (grey) was monitored by RelA IP of NE at mentioned time intervals after TNFα treatment. (Bottom panel) The RelA:CBP/p300 interaction at various time intervals after TNFα stimulation was monitored by Co-IP experiments using CBP/p300 antibodies for RelA(wt) (black) and RelA(Ser467Ala) mutant (red) (from Figure 4E). The binding curve for RelA(wt) is shifted to the left, where the concentration of p-Ser467–RelA is highest. The RelA(Ser467Ala) mutant shows a linear change in the aforementioned binding with respect to the concentration of total nRelA. The lines joining the data points are only used for the purpose of viewing clarity. (TIF) [file pbio.1001647.s006.tif]

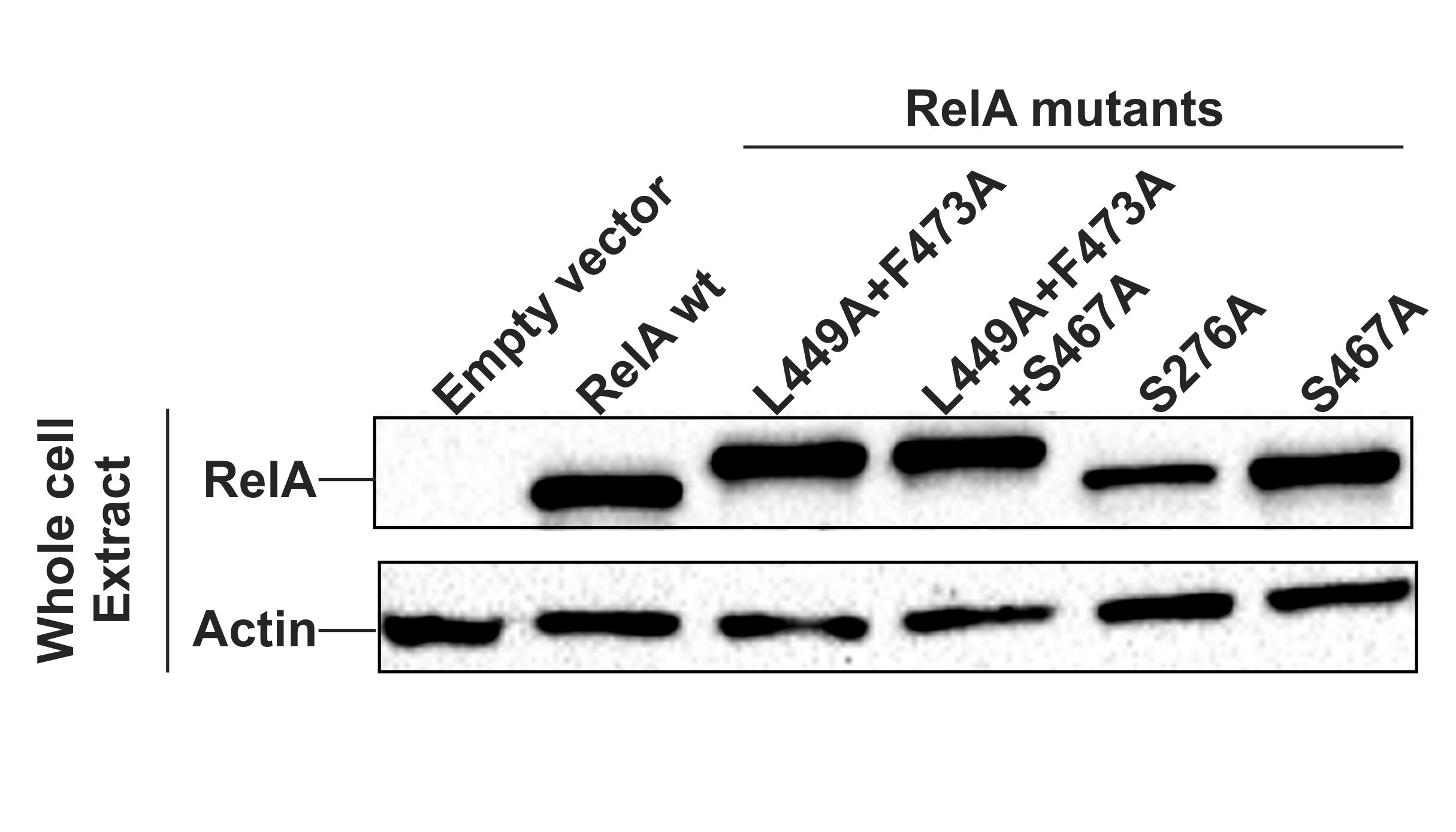

Supplement: Figure S7 — Expression levels of RelA in the RelA reconstituted rela −/− cells. RelA protein levels (wt and mutants) were determined in WEs of the RelA reconstituted rela −/− cell lines detected by immunoblotting of RelA. Actin was used as the loading control. (TIF) [file pbio.1001647.s007.tif]

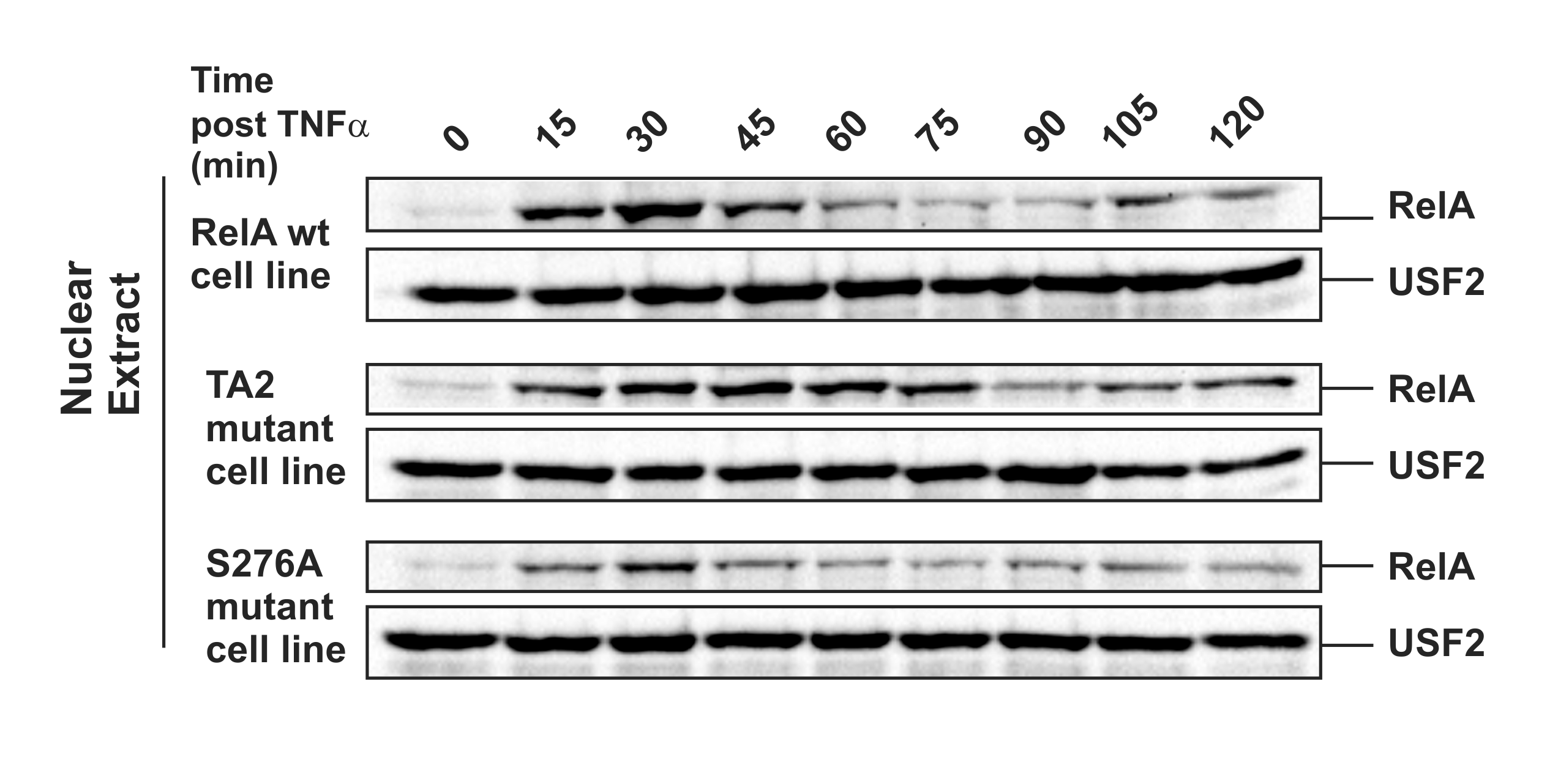

Supplement: Figure S8 — The nuclear residence time of the RelA(TA2) mutant is longer than RelA(wt) and RelA(Ser276Ala). RelA nuclear translocation assay in RelA(wt/mutants) reconstituted rela −/− cells following stimulation with 5 ng/ml TNFα. Upstream stimulatory factor 2 (USF2) was used as the loading control for the NEs. (TIF) [file pbio.1001647.s008.tif]

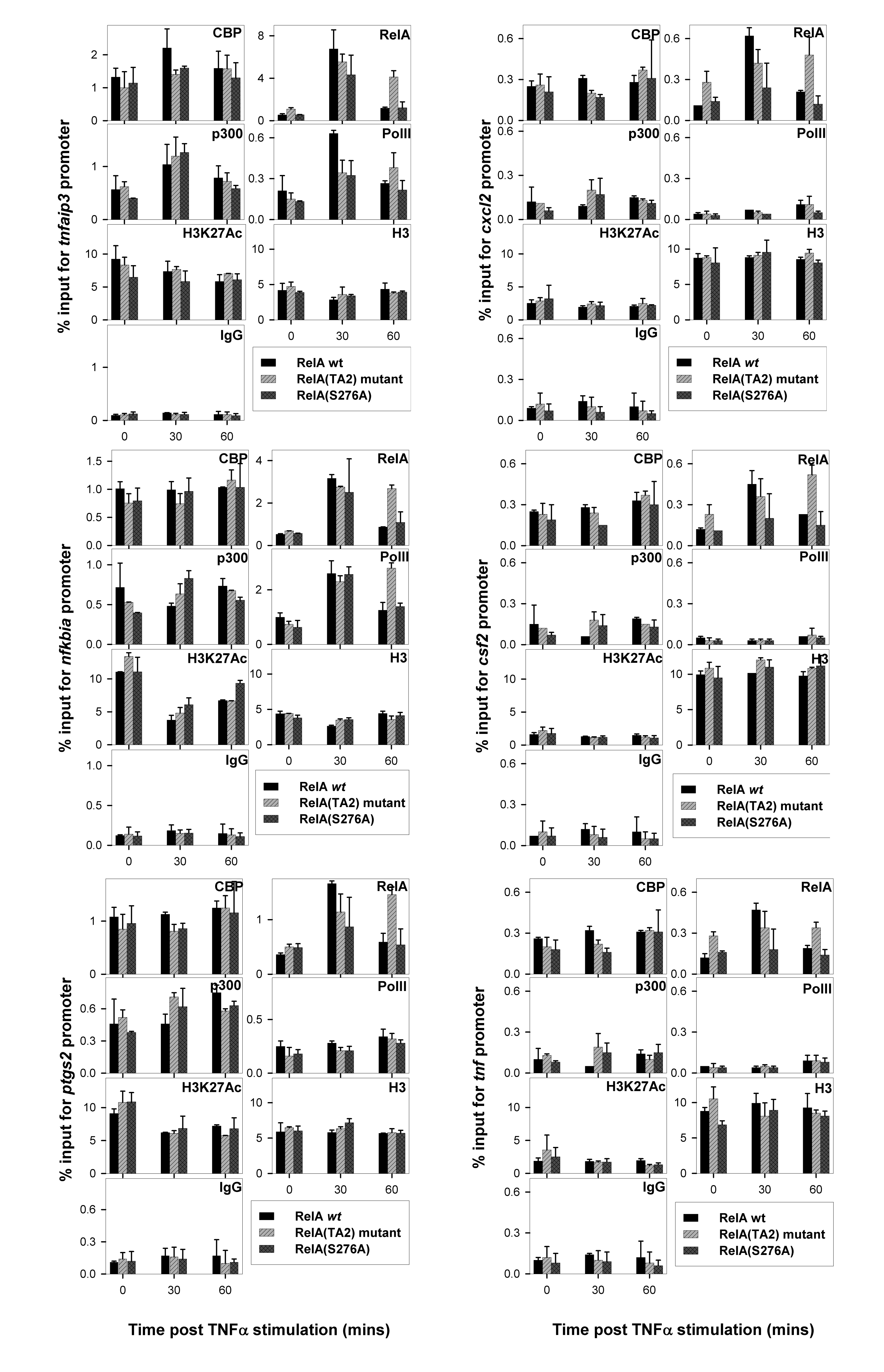

Supplement: Figure S9 — CBP/p300 aids recruitment of RelA to its target promoters. ChIP experiments were performed with RelA, CBP, p300, H3, H3K27ac, RNA polymerase II, and IgG antibodies in RelA(wt), RelA(TA2), or RelA(Ser276Ala) reconstituted rela −/− cells under unstimulated or stimulated conditions for 30 min or 60 min with TNFα. Results are presented as average %input values with standard deviations from two independent chromatin preparations. The respective gene names are mentioned on the y-axis of each group of panels. (TIF) [file pbio.1001647.s009.tif]

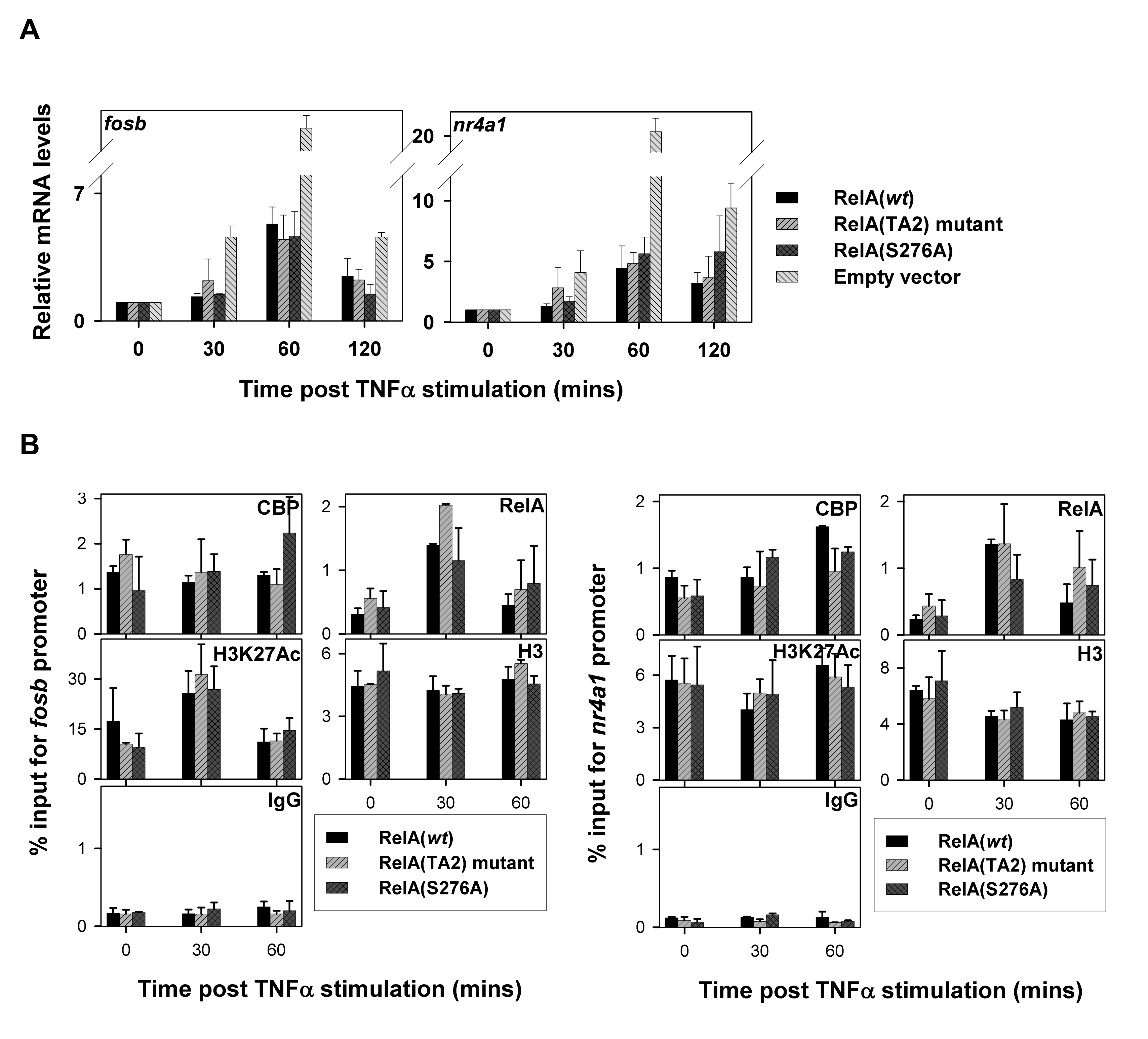

Supplement: Figure S10 — RelA(TA2) mutant has normal promoter recruitment for RelA:CBP/p300 interaction-independent RelA-regulated genes. (A) Gene expression profile of fosb and nr4a1 genes. fosb (NM_008036) and nr4a1 (NM_010444) genes belong to the Group D genes (Figure 5B). Kasper et al. have shown both nr4a1 and fosb were highly expressed following forskolin stimulation in cbp −/− and p300 −/− MEF cells [75], thus indicating their CBP/p300-independent expression. Here both the genes showed higher TNFα-induced expression in empty vector reconstituted rela −/− cells compared to the RelA(wt/mutant) reconstituted cells. The level of TNFα-induced expression of nr4a1 and fosb was reduced in the presence of RelA(wt) as well as mutants to a similar extent. This suggests that the transcription of both the genes is regulated by RelA but is independent of RelA:CBP/p300 interaction. (B) RelA(wt), RelA(TA2) mutant, and RelA(S276) mutant are recruited to a similar extent on the promoters of fosb and nr4a1. ChIP-qPCR experiments were performed as mentioned earlier (Figure 7) to study RelA recruitment on the promoter sites of fosb and nr4a1. (TIF) [file pbio.1001647.s010.tif]

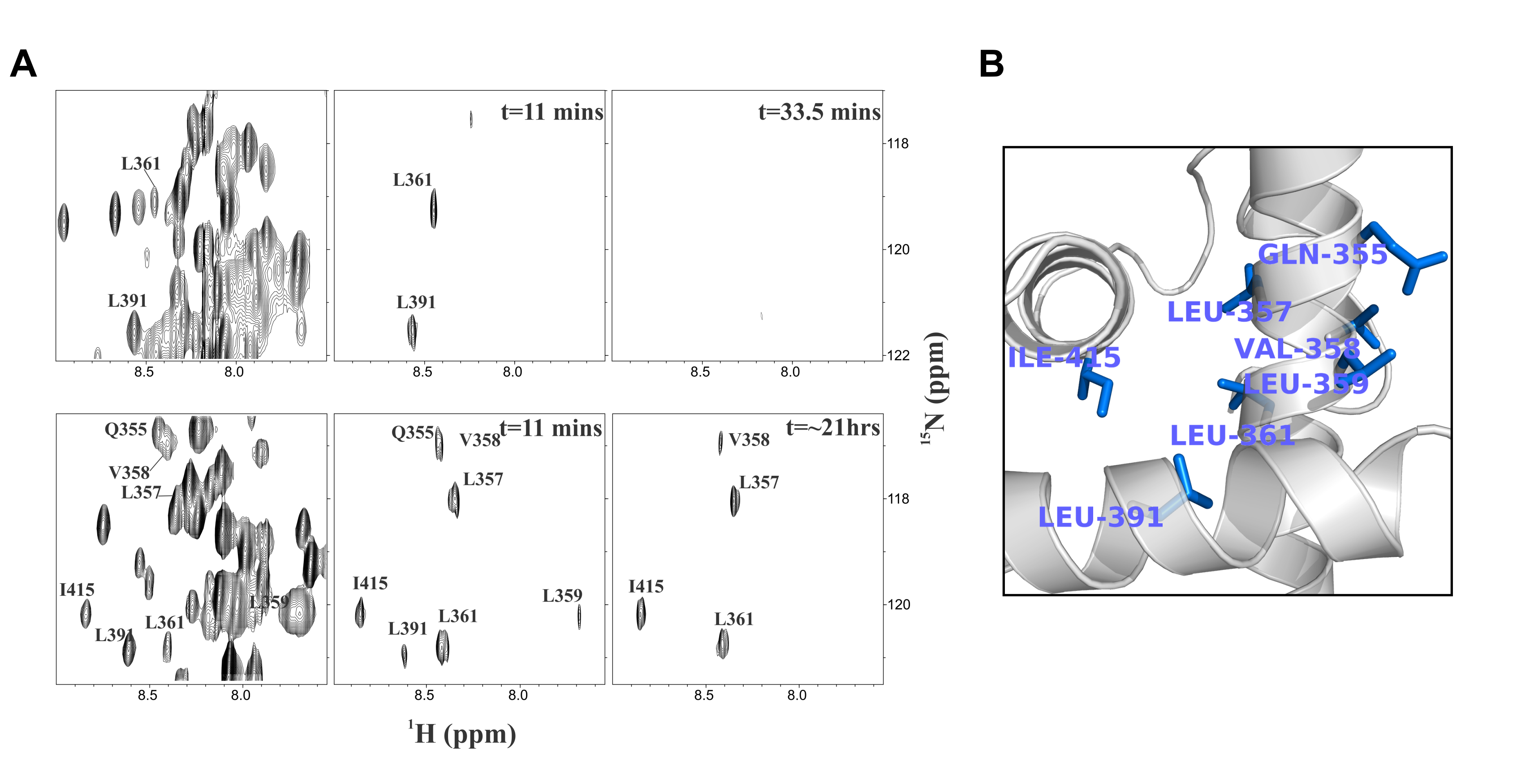

Supplement: Figure S11 — The hydrophobic core of TAZ1 is stabilized upon binding to RelA–TA2 as seen from the deuterium exchange experiments of 15N-labeled TAZ1 and 15N-labeled TAZ1 in complex with unlabeled RelA–TA2. (A, Top row) 2D [15N–1H]-HSQC spectrum of free TAZ1 in H2O (left panel), 11 min after addition of D2O (middle panel), and about 33 min (right panel) after addition of D2O. (Bottom row) 2D [15N–1H]-HSQC spectrum of TAZ1 in complex with unlabeled RelA–TA2 in H2O (left panel), 11 min after addition of D2O (middle panel), and about 21 h (right panel) after addition of D2O. The time reported here is of the first free induction decay (fid) recorded after addition of D2O. (B) Cartoon representations of TAZ1 with amino acid residues observed in the 2D [15N–1H]-HSQC spectrum post-D2O exchange with higher resistance to exchange are shown as blue sticks. The residues with higher resistance toward D2O exchange in TAZ1 (in complex with RelA–TA2) belong either to the hydrophobic core (Leu357, Leu391, and Ile415) or around it (Leu360, Gln355, Val358, Leu359, and Leu361). (TIF) [file pbio.1001647.s011.tif]
