# Supplementary material for: Analysis of the RelA:CBP/p300 Interaction Reveals Its Involvement in NF-κB-Driven Transcription
Source: PLoS Biol. 2013 Sep 3;11(9):e1001647. doi: 10.1371/journal.pbio.1001647 (PMC3760798; doi:10.1371/journal.pbio.1001647)
Supplement: Table S1 — Parameters for the ODE-based model for NF-κB regulation. (DOC) [file pbio.1001647.s012.doc]

**Table S1:** Parameters for the ODE-based model for NF-κB regulation

| Value | Description | Comments |
| --- | --- | --- |
| 0.33 min^-1^ | mRNA degradation IκB-α/β/ε | 2x original value in (corresponds to a more realistic half-life of ~20 min |
| 5.2 x10^-4^ min^-1^ | NF-κB independent mRNA production IκBα | Compensates for reduced mRNA stability |
| (6/0.3/0.2)10^-2^ min^-1^ | Rate constant IκBα mRNA production (WT/TA2/Ser**^276^**Ala) |  |
| 4 x10^-3^ min^-1^ | Rate constant IκBε mRNA production | Produced wt IκBε dynamics similar to those in |
| K_A_->0.2 mM  J->5.8 K_B_T  L->-1.3 K_B_T  N->0.1 K_B_T | Promoter activation parameters for IκBε | As in |
| K_A_->0.2 mM  J->5.8 K_B_T  L->-1.3 K_B_T  N->0.1 K_B_T | Promoter activation parameters for IκBα | As in |

1. Werner SL, Barken D, Hoffmann A (2005) Stimulus specificity of gene expression programs determined by temporal control of IKK activity. Science 309: 1857-1861.

2. Giorgetti L, Siggers T, Tiana G, Caprara G, Notarbartolo S, et al. (2010) Noncooperative interactions between transcription factors and clustered DNA binding sites enable graded transcriptional responses to environmental inputs. Mol Cell 37: 418-428.
