# Supplementary material for: Analysis of the RelA:CBP/p300 Interaction Reveals Its Involvement in NF-κB-Driven Transcription
Source: PLoS Biol. 2013 Sep 3;11(9):e1001647. doi: 10.1371/journal.pbio.1001647 (PMC3760798; doi:10.1371/journal.pbio.1001647)
Supplement: Table S2 — Changes in IKK activity induced by exposure to TNFα. (DOC) [file pbio.1001647.s013.doc]

**Table S2: Changes in IKK activity induced by exposure to TNFα**

| Time (min) | 0’ | 7’ | 12’ | 17’ | 20’ | 25’ | 35’ | 45’ | 55’ | 60’ | 80’ | 360’ |
| --- | --- | --- | --- | --- | --- | --- | --- | --- | --- | --- | --- | --- |
| IKK activity multiplier | 5 | 40 | 70 | 90 | 100 | 90 | 65 | 50 | 36 | 30 | 30 | 30 |
