# Supplementary material for: Analysis of the RelA:CBP/p300 Interaction Reveals Its Involvement in NF-κB-Driven Transcription
Source: PLoS Biol. 2013 Sep 3;11(9):e1001647. doi: 10.1371/journal.pbio.1001647 (PMC3760798; doi:10.1371/journal.pbio.1001647)
Supplement: Text S1 — Supporting procedures legends, deuterium exchange of backbone amide protons, and gel filtration. (DOC) [file pbio.1001647.s014.doc]

**Text S1 Supplementary Procedures**

**Deuterium exchange of backbone amide protons.** Approximately 500 μl of 1 mM ^15^N-labeled TAZ1 in complex with an excess of unlabeled RelA-TA2 fragment was loaded on to a 2 ml Desalting column (Thermoscientific) preequilibrated with D_2_O buffer (10 mM Tris (pD 6.5), 40 mM NaCl and 2 mM DTT). The sample was centrifuged for 2 min at 1000g and immediately loading onto a pre-shimmed NMR spectrometer (Bruker DRX600). A series of [^15^N-^1^H] HSQC spectra were then recorded at the time points in Figure S9.

**Gel Filtration.** Protein samples in 20 mM Tris (pH 7.5), 50 mM NaCl and 2 mM DTT were applied to Superdex 75^TM^ HR 10/30 gel permeation column (GE Life Sciences), equilibrated in the same buffer and connected to the Äkta purifier. The elution profile of the column was calibrated using Biorad standard (# 151-1901) for molecular weight determination.
